# β-Hydroxybutyrate ameliorates lipopolysaccharide-induced liver injury through β-hydroxybutyrylation of the SOD2 protein in mice

**DOI:** 10.1016/j.redox.2025.103949

**Published:** 2025-11-27

**Authors:** Ya-Ping Bai, Yan Zhang, Zhi-Yuan Chen, Kai Li, De-Guo Wang, Shu-Jun Wan, Cui-Wei Zhang, Yue Sun, Zhi-Chao Li, Kun Lv, Lei Zha, Xiang Kong

**Affiliations:** a Department of pharmacy, Anhui College of Traditional Chinese Medicine, Wuhu, 241003, China; b Anhui Provincial Key Laboratory of Non-coding RNA Basic and Clinical Transformation, The First Affiliated Hospital of Wannan Medical College, Wuhu, 241001, China; c Department of Gastroenterology, The First Affiliated Hospital of Wannan Medical College, Wuhu, 241001, China; d Department of Gerontology, Geriatric endocrinology unit, The First Affiliated Hospital of Wannan Medical College, Wuhu, 241001, China; e National Clinical Research Center for Geriatric Diseases, Anhui Provincial Sub-center, Wuhu, 241001, China; f Department of Respiratory Medicine, The First Affiliated Hospital of Wannan Medical College, Wuhu, 241001, China

**Keywords:** β-Hydroxybutyrate (β-OHB), Lysine β-hydroxybutyrylation (kbhb), Superoxide dismutase 2 (SOD2), NLRP3 inflammasome, Apoptosis

## Abstract

Septic liver injury is a major complication of sepsis, driven in part by oxidative stress–induced macrophage inflammasome activation and hepatocyte apoptosis. β-Hydroxybutyrate (β-OHB), a key ketone body, induces lysine β-hydroxybutyrylation (Kbhb), a novel post-translational modification, yet its role in septic liver injury remains unclear. In this study, we demonstrated that β-OHB markedly increased Kbhb modification of superoxide dismutase 2 (SOD2), a mitochondrial antioxidant enzyme, in both macrophages and hepatocytes. Mechanistically, β-OHB promoted Kbhb at lysine 68 of SOD2, thereby preventing ubiquitin–proteasome–mediated degradation, and then stabilizing the protein, which enhanced its enzymatic activity, and reduced mitochondrial reactive oxygen species accumulation. Consequently, during lipopolysaccharide-induced septic liver injury, SOD2 Kbhb suppressed NLRP3 inflammasome activation in macrophages and protected hepatocytes from apoptosis. Collectively, our findings identify the β-OHB–SOD2–Kbhb axis as a previously unrecognized antioxidant pathway and highlight its therapeutic potential in sepsis.

## 1 Introduction

Sepsis is a life-threatening condition characterized by systemic inflammation and multi-organ failure, with the liver among the most commonly affected organs [1]. Liver dysfunction can occur in the early stage of sepsis and is caused by endotoxemia, cytokine storms and oxidative stress, leading to microcirculatory disturbances, and hepatocyte damage [2]. Early liver impairment significantly increases mortality and serves as an independent predictor of poor prognosis in patients with sepsis [3].

β-Hydroxybutyrate (β-OHB), the primary ketone body produced by the liver, functions not only as an alternative energy substrate for other tissues under certain conditions but also as a key signaling molecule and epigenetic regulator [4]. Increasing evidence shows that circulating β-OHB levels are inversely associated with the severity of sepsis-induced liver injury [5,6]. Ketone ester (KE, also known as BD-AcAc2) is an exogenous ketone compound that rapidly elevates β-OHB levels following oral administration [7]. In individuals with obesity, 14-days KE supplementation suppresses lipopolysaccharide (LPS)-induced caspase-1 activity in human monocytes [8]. Additionally, KE has been shown to reduce LPS-induced systemic inflammation and liver injury in mice [9], suggesting a protective role of β-OHB in sepsis-related liver damage. However, the underlying molecular mechanisms remain to be elucidated.

Recent studies have demonstrated that excessive production of reactive oxygen species (ROS) plays a key role in the pathogenesis of sepsis-induced liver injury [[10], [11], [12], [13], [14]]. Notably, β-OHB exhibits antioxidant properties in both *in vivo* and *in vitro* moedels [15], and has been shown to reduce LPS-induced oxidative damage in mouse myocardium [16]. These findings suggest that BHB might alleviate sepsis-related liver injury, at least partially, by reducing ROS production.

Lysine β-hydroxybutyrylation (Kbhb), a post-translational modification (PTM) mediated by β-OHB, is essential for modulating the expression and activity of both histone and non-histone proteins [17]. Kbhb modification has been implicated in diverse physiological and pathological processes, including metabolic adaptation to starvation [18], inhibition of M1 macrophage polarization [19], alleviation of heart failure [20], suppression of tumor angiogenesis and metastasis [21,22], and mitigation of alcohol-induced liver injury [23].

Nevertheless, it remains unclear whether Kbhb modification directly contributes to the antioxidant effects of β-OHB. Therefore, the present study aimed to investigate the role of Kbhb modification in mediating the antioxidant functions of β-OHB and to determine its impact on hepatic injury in the context of sepsis.

## 2 Materials and methods

### 2.1 Cell lines, culture conditions and treatment

The mouse J774A.1 macrophage (#CC9041) and HEK293T cell line were purchased from Cellcook (Guangzhou, China). The mouse AML12 hepatocyte line was purchased from Procell (#CL-0602, Wuhan, China). These cell lines were authenticated and tested for mycoplasma contamination, and have been used in our previous studies [19,24]. The J774A.1 and HEK293T cells were cultured in Dulbecco's modified Eagle medium (DMEM, #C11995500BT, Gibco, New York, USA), supplemented with 10 % fetal bovine serum (FBS, #10099141C, Gibco). The AML12 hepatocytes were maintained in DMEM supplemented with 10 % FBS, 10 μg/ml insulin, 5.5 μg/ml transferrin and 5 ng/ml selenium. The β-OHB (#298360) and LPS (#L2880) were purchased from Sigma (Missouri, USA), while the cycloheximide (CHX, #HY-12320) and the proteasome inhibitor MG132 (#HY-13259) were obtained from MedChemExpress (Shanghai, China). The mitochondrial fractions from HEK293T cells were isolated using a Cell Mitochondrial Isolation Kit (#C3601, Beyotime, Shanghai, China). The treatment durations and agent concentrations were detailed in the figures and/or their corresponding legends.

### 2.2 Construction of mutant plasmids

The mouse pCMV-3 × FLAG-superoxide dismutase 2 (SOD2)-Neo plasmid (#P72394) was purchased from MiaoLingBio (Wuhan, China). Site-directed mutation plasmids of SOD2 (K68R, K130R and K134R) were constructed from the wild-type (WT) Flag-SOD2 plasmid using the Mut Express II Fast Mutagenesis Kit (#C214, Vazyme, Nanjing, China). The generated mutant plasmids were validated through Sanger sequencing and aligned with the reference sequence (RefSeq accession number NM_013671.3). The primers used to generate the site-directed mutation plasmids are listed in Table 1. The HEK293T, J774A.1 and AML12 cells were transfected with the mutant plasmids using the GenMute™ siRNA&DNA Transfection reagent (#SL100568, SignaGen, Gaithersburg, USA) and GenMute™ Transfection buffer (#SL100572, SignaGen).

**Table 1:** The primer sequences used for generating SOD2 site-directed mutation plasmids.

Genes	Primers	Primer sequences (5' → 3′)
SOD2-K68R	Forward	GAGGAGAGGTACCACGAGGCTCTGGCCAAGGG
	Reverse	TCGTGGTACCTCTCCTCGGTGGCGTTGAGATT
SOD2-K130R	Forward	GGGTCTTTTGAGAGGTTTAAGGAGAAGCTGACAGCCG
	Reverse	AACCTCTCAAAAGACCCAAAGTCACGCTTGAT
SOD2-K134R	Forward	TAAGGAGAGGCTGACAGCCGTGTCTGTGGGAG
	Reverse	CTGTCAGCCTCTCCTTAAACTTCTCAAAAGACCC

### 2.3 Immunoblotting analysis

Total proteins were separated by sodium dodecyl sulphate–polyacrylamide gel electrophoresis (SDS–PAGE) and transferred onto polyvinylidene fluoride (PVDF) membranes. The membranes were incubated overnight with the following primary antibodies: anti-SOD2, anti-Kbhb, anti-Flag, anti-ubiquitin, anti-NOD-like receptor family pyrin domain containing 3 (NLRP3), anti-apoptosis-associated speck-like protein containing a CARD (ASC), anti-cleaved caspase-1, anti-B-cell lymphoma 2 (Bcl-2) and anti-Bcl-2-associated X protein (Bax). After primary antibody incubation, the membranes were washed and subsequently incubated with the appropriate secondary antibodies for 2 h. The antibody-bound proteins were visualised using an enhanced chemiluminescence (ECL) kit (#MK042A, BIOMIKY, Shanghai, China), and the band intensities were quantified using the ImageJ software. The antibodies against SOD2 (#A1340), Flag (#AE092), ubiquitin (#A19686), NLRP3 (#A24294), ASC (#A22046), Bcl-2 (#A19693) and Bax (#A19684) were purchased from Abclonal (Wuhan, China). The anti-Kbhb antibody (#PTM-1201RM) was purchased from PTM BioLabs (Hangzhou, China). The anti-cleaved-caspase 1 antibody (#AF4005) was purchased from Affinity (Liyang, China). The anti-COX IV antibody (#ABL1060) was purchased from Abbkine (Wuhan, China).

### 2.4 Co-immunoprecipitation (Co-IP) analysis

Endogenous Co-IP assays were carried out using the Pierce™ Classic Magnetic IP/Co-IP kit (#88804, Thermo Fisher Scientific, Massachusetts, USA) with anti- SOD2 and IgG (control) antibodies, while exogenous Co-IP assays were conducted using the ChainFree™ Flag-Tag Co-IP Kit (#FI8801, Fitgene, Guangzhou, China). The bound proteins were eluted, and the supernatants were collected for immunoblotting analysis of Kbhb, SOD2 and ubiquitin. Clean-Blot IP Detection Reagent (HRP) (#21230, Thermo Fisher Scientific) was used to was used to minimise interference from the immunoglobulin heavy and light chains.

### 2.5 Measurement of mitochondrial ROS

The production of mitochondrial ROS in J774A.1 and AML12 cells was evaluated using MitoSOX™ Red (#S0061S, Beyotime), a fluorogenic dye specifically targeting mitochondrial superoxide. Cells from each experimental group were incubated with 5 μM MitoSOX™ Red at 37 °C for 15 min, followed by three washes with PBS. Fluorescence signals were recorded using a Zeiss LSM 780 confocal microscope (Oberkochen, Germany) and a Cytomics™ FC500 flow cytometer (Beckman, California, USA). The mean fluorescence intensity (MFI) of MitoSOX™ Red was subsequently quantified to assess mitochondrial ROS levels.

### 2.6 Evaluation of cell apoptosis by flow cytometric analysis and caspase-3 staining

For flow cytometric analysis, AML12 cells were were co-stained with 5 μl Annexin V-FITC/propidium iodide (PI, #G1511, Servicebio, Wuhan, China) for 15 min in the dark. Stained cells were analyzed on a Cytomics™ FC500 flow cytometer (Beckman), and the apoptotic rate was calculated as the proportion of Annexin V-positive cells relative to the total cell population.

To further evaluate apoptosis, the GreenNuc™ caspase-3 Detection kit (#C1168S, Beyotime) was employed. AML12 cells were incubated with 5 μM GreenNuc caspase-3 substrate in fresh medium at room temperature for 30 min, protected from light. Hoechst 33342 dye (#C1027, Beyotime) was then added (5 μl per well) and incubated for an additional 5 min. Active caspase-3 expression was visualised as green fluorescence in the nucleus, and images were captured using a Zeiss LSM 780 confocal microscope (Oberkochen, Germany).

### 2.7 Animal experiments

The experimental procedures in this study were reviewed and approved by the Animal Ethics Committee of Wannan Medical College (Approval No. WNMC-AWE-2024156, Anhui, China). Eight-week-old male C57BL/6 mice (GemPharmatech Co., Nanjing, China) were housed under specific pathogen-free conditions and acclimatised for one week prior to experimentation. Mice were maintained under a controlled 12-h light/dark cycle at a temperature of 22–26 °C.

To investigate the protective effect of β-OHB against the sepsis-induced liver injury *in vivo*, the mice were randomly assigned into three groups (n = 6 per group): control (Con), LPS-treated (LPS) and LPS with KE pre-treatment (LPS + KE).

To further assess the role of SOD2 Kbhb modification in the protective effect of β-OHB on the sepsis-induced liver injury, we constructed an adeno-associated virus serotype 8 (AAV8) vector carrying either the wild-type mouse SOD2 gene (AAV8-SOD2-3 × FLAG-Zsgreen1) or the mutant SOD2 gene (AAV8-SOD2-K68R- 3 × FLAG-Zsgreen1). All the AAV8 vectors were produced by Obio Technology Co., Ltd. (Shanghai, China). Subsequently, the mice were randomly assigned to four groups (n = 6 per group): Group 1, AAV8-SOD2 + LPS; Group 2, AAV8-SOD2 + LPS + KE; Group 3, AAV8-SOD2-K68R + LPS; Group 4, AAV8-SOD2-K68R + LPS + KE. Each mouse received a tail vein injection of 100 μl viral suspension containing 2 × 10^11^ AAV8 vector genomes [24]. Two weeks after transduction, the mice were subjected to the KE and/or LPS treatment as per their group assignment.

The mice in the KE pre-treatment group received KE (3 mg/g/d) via oral gavage at 10:00 a.m. for three consecutive days. This dosage was selected based on previous reports [9,16], which demonstrated that 3 mg/g/d of KE effectively elevates circulating β-OHB levels without causing ketoacidosis. Following the final KE administration, LPS (5 mg/kg) was delivered intraperitoneally to induce sepsis [19]. No mortality was recorded within 24 h after LPS administration. After this period, the serum and liver tissues were harvested for subsequent analyses.

### 2.8 Biochemical analysis and enzyme-linked immunosorbent assay (ELISA)

The β-OHB concentrations in mice tail blood were determined using a ketone meter (#FreeStyle Optium Neo, Abbott, Oxon, UK). The activity of SOD2 in cultured cells and mouse liver tissues was assessed via the WST-8 method (#S0103, Beyotime). Caspase-1 enzymatic activity in J774A.1 cells was measured using a colourimetric caspase-1 activity assay kit (#C1102, Beyotime). The serum levels of alanine aminotransferase (ALT) and aspartate aminotransferase (AST) were quantified using enzymatic colourimetric methods (C009-2-1 and C010-2-1, Jiancheng Bioengineering Institute, Nanjing, China). Interleukin-1β (IL-1β) levels in the supernatants of J774A.1 cells and in mouse liver tissue homogenates were analyzed using a cytokine-specific ELISA kit (#RK00006, Abclonal).

### 2.9 Histological staining

The liver tissue samples were fixed in 10 % neutral-buffered formalin for 24 h, embedded in paraffin, sectioned at a thickness of 4 μm, and subsequently processed for H&E staining.

### 2.10 Statistical analysis

The data were expressed as the mean ± standard deviation. Statistical differences among multiple groups were assessed using one-way analysis of variance, followed by Tukey's multiple comparison test. Comparisons between two groups were performed using the two-tailed Student's *t*-test. The *P*-value <0.05 was considered statistically significant.

## 3 Results

### 3.1 β-OHB-mediated kbhb modification of the SOD2 protein

**Fig. 1:**
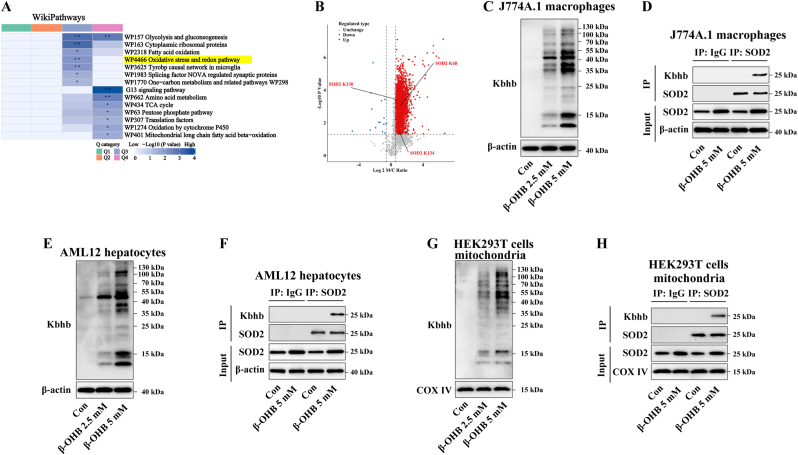
β-OHB-mediated Kbhb modification of the SOD2 protein. (A) WikiPathways enrichment **analysis of** proteins with **upregulated** kbhb sites **in** β-OHB-incubated mouse BMDMs. (B) Volcano plot showing significantly different Kbhb sites between β-OHB-treated and control BMDMs. (C) J774A.1 cells were treated with β-OHB at the indicated concentrations for 24 h. Immunoblotting analysis of the Kbhb modification in the J774A.1 cells (n = 3). (D) J774A.1 cells were treated with or without 5 mM β-OHB for 24 h. Co-IP analysis of the SOD2 Kbhb levels in J774A.1 cells (n = 3). (E) AML12 cells were treated with β-OHB at the indicated concentrations for 24 h. Immunoblotting analysis of the Kbhb modification in the AML12 cells (n = 3). (F) AML12 cells were treated with or without 5 mM β-OHB for 24 h. Co-IP analysis of the SOD2 Kbhb levels in AML12 cells (n = 3). (G) HEK293T cells were treated with β-OHB at the indicated concentrations for 24 h. Mitochondria were isolated and analyzed for Kbhb modification by immunoblotting (n = 3). (n = 3). (H) HEK293T cells were treated with or without 5 mM β-OHB for 24 h. Mitochondria were isolated and analyzed for the SOD2 Kbhb levels by Co-IP (n = 3).

Our recent research demonstrated that β-OHB induces Kbhb modification of proteins in macrophages [19]. Global proteomic analysis identified 3469 upregulated differential Kbhb sites across 1549 proteins in β-OHB-incubated mouse bone marrow-derived macrophages (BMDMs), compared to the control BMDMs [19]. Subsequent bioinformatic analysis, WikiPathways enrichment, revealed that Kbhb-modified proteins were predominantly associated with the oxidative stress and redox pathway (Fig. 1A). Within this pathway, 22 proteins were found to undergo Kbhb modification (Table S1). Among these modified proteins, SOD2, a mitochondrial antioxidant enzyme, exhibited significant Kbhb modification (Fig. 1B).

Consistent with our previous findings in mouse RAW264.7 macrophages and BMDMs [19], immunoblotting analysis revealed that treatment with 2.5 or 5 mM β-OHB (at concentrations comparable to the circulating β-OHB levels observed in individuals following a ketogenic diet or receiving KE administration [25]) for 24 h markedly increased Kbhb modification in J774A.1 cells (Fig. 1C). Furthermore, Co-IP analysis confirmed that β-OHB treatment significantly enhanced the Kbhb modification of the SOD2 protein in J774A.1 cells (Fig. 1D).

Beyond macrophages, β-OHB has also been shown to promote the global protein Kbhb modification in hepatocytes [18,23]. For instance, starvation (48 h fasting), which elevates the circulating β-OHB levels and increase Kbhb modification in the mouse liver [18]. Notably, proteomic analysis using mass spectrometry (MS) of liver tissues from starved mice revealed significant Kbhb modification of the SOD2 protein [18]. In line with these findings, our results demonstrated that 2.5 or 5 mM β-OHB treatment for 24 h markedly increased Kbhb modification in AML12 cells (Fig. 1E). Likewise, β-OHB significantly enhanced the Kbhb modification of SOD2 in AML12 cells (Fig. 1F). Altogether, these results suggest that β-OHB promotes Kbhb modification of the SOD2 protein in both macrophages and hepatocytes.

Previous studies, including ours, have shown that β-OHB induces Kbhb modification of mitochondrial proteins [18,19]. Consistent with these findings, treatment with 2.5 or 5 mM β-OHB for 24 h increased Kbhb modification in the mitochondria of HEK293T cells (Fig. 1G). As shown in Fig. 1H, β-OHB significantly enhanced the Kbhb modification of SOD2 in the mitochondria of HEK293T cells.

### 3.2 K68 as the primary functional site of kbhb modification on the SOD2 protein

**Fig. 2:**
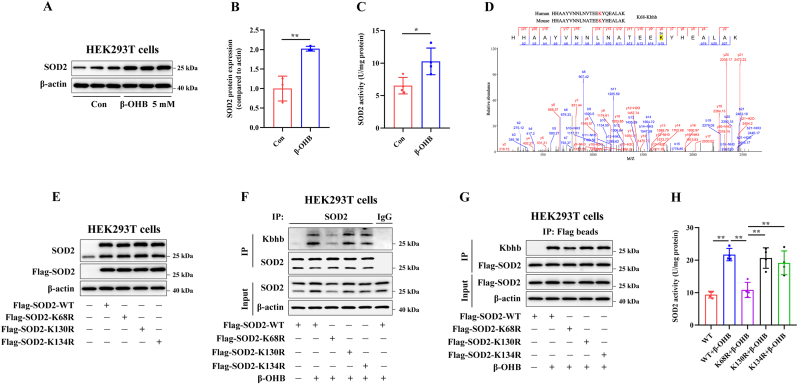
K68 as the primary functional site of Kbhb modification on the SOD2 protein. HEK293T cells were treated with 5 mM β-OHB for 24 h. (A,B) Immunoblotting analysis of the protein expression of SOD2 (n = 3). (C) The intracellular enzymatic activity of SOD2 was measured (n = 4). (D) MS data showing SOD2 Kbhb modification sites (K68) identified in β-OHB-treated BMDMs. Top: conservation of Kbhb sites in human and mouse SOD2 proteins. (E) The overexpression efficiency of the Flag-SOD2-WT or -K to R mutant plasmids was assessed. (F, G) HEK293T cells were transfected with the indicated plasmids and treated with 5 mM β-OHB for 24 h, followed by immunoprecipitation using anti-SOD2 antibodies (F) or anti-Flag beads (G). Immunoblotting analysis of the Kbhb levels of SOD2 in HEK293T cells (n = 3). (H) The intracellular enzymatic activity of SOD2 was measured (n = 4). ∗*p* < 0.05, ∗∗*p* < 0.01.

As shown in Fig. 1D and F, treatment with 5 mM β-OHB for 24 h clearly increased the expression of the SOD2 protein in the whole-cell lysates (labelled as 'input' in the figure) of J774A.1 and AML12 cells. The same β-OHB treatment also significantly increased SOD2 protein expression and enzymatic activity in HEK293T cells (Fig. 2A–C). MS data identified three potential Kbhb modification sites on the SOD2 protein in β-OHB-treated BMDMs (Fig. 2D–SFig. 1A and B): lysine 68 (K68), lysine 130 (K130) and lysine 134 (K134), which were consistent with the MS data obtained from liver tissues of starved mice [18]. Furthermore, sequence analysis of the SOD2 protein revealed homologous sequences near these amino acid residues in both humans and mice, indicating that the Kbhb sites on SOD2 protein are conserved (Fig. 2D–SFig. 1A and B).

HEK293T cells were transfected with Flag-SOD2-WT or -mutant plasmids [in which lysine residues (K) were replaced with arginine (R)], followed by immunoprecipitation using anti-SOD2 antibodies (Fig. 2F) or anti-Flag beads (Fig. 2G). The overexpression efficiency of SOD2 plasmids is shown in Fig. 2E. The results revealed that the SOD2-K68R mutant had significantly lower β-OHB-induced SOD2 Kbhb levels, along with decreased SOD2 expression and enzymatic activity, compared with the WT and other SOD2 mutants (Fig. 2F–H), suggesting that K68 is the primary functional Kbhb site on the SOD2 protein.

### 3.3 Kbhb modification at the SOD2 K68 site stabilizes the SOD2 protein

**Fig. 3:**
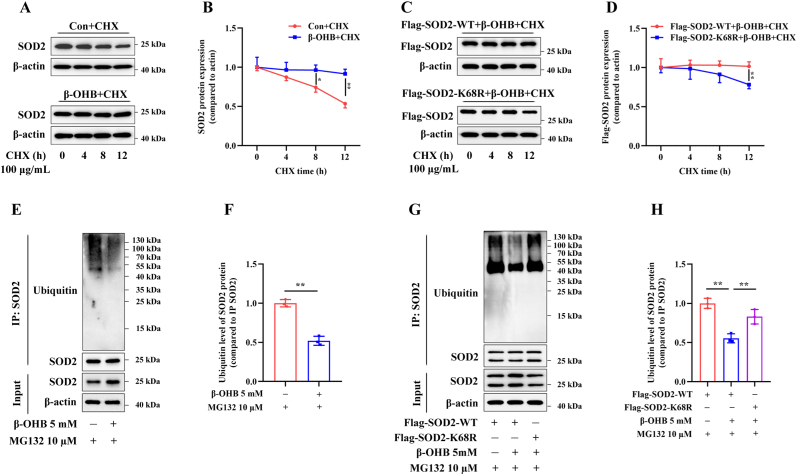
**Kbhb modification of the SOD2 K68 site stabilizes the SOD2 protein**. (A,B) HEK293T cells were treated with or without 5 mM β-OHB for 24 h, followed by incubation with 100 μg/mL CHX for the indicated times. Immunoblotting analysis of the SOD2 protein expression (n = 3). (C,D) HEK293T cells were transfected with the indicated plasmids, treated with 5 mM β-OHB for 24 h, and then exposed to 100 μg/mL CHX for the indicated times. Immunoblotting analysis of the Flag-tagged protein expression (n = 3). (E,F) HEK293T cells were treated with or without 5 mM β-OHB for 24 h, then incubated with 10 μM MG132 for 12 h, followed by immunoprecipitation using anti-SOD2 antibodies. The histogram represents the ubiquitin level of SOD2 protein (n = 3). (G,H) HEK293T cells were transfected with the indicated plasmids and treated with or without 5 mM β-OHB for 24 h, then exposed to 10 μM MG132 for 12 h, followed by immunoprecipitation using anti-SOD2 antibodies. The histogram represents the ubiquitin level of SOD2 protein (n = 3). ∗*p* < 0.05, ∗∗*p* < 0.01.

Subsequently, we explored the potential mechanisms through which Kbhb modification enhances SOD2 expression. Kbhb modification induced by β-OHB has previously been shown to stabilize proteins such as Snail and SLC25A5 [22,26]. We therefore examined whether β-OHB exerts a similar effect on SOD2. HEK293T cells were treated with 100 μg/mL CHX to inhibit protein synthesis, and 5 mM β-OHB administration was found to significantly delay the SOD2 degradation (Fig. 3A and B). Moreover, transfection with the SOD2-K68R plasmid notably compromised protein stability (Fig. 3C and D), suggesting that Kbhb modification at K68 contributes to the β-OHB-induced increase in SOD2 expression.

To further investigate the mechanisms underlying Kbhb modification and SOD2 protein stability, HEK293T cells were exposed to the proteasome inhibitor MG132. Immunoblotting revealed accumulation of SOD2 in 10 μM MG132-treated cells (SFig. 2A and B), further indicating that SOD2 degradation is regulated by the ubiquitin–proteasome pathway [27,28]. Cellular ubiquitination assays subsequently showed a significant reduction in the SOD2 ubiquitination following 5 mM β-OHB treatment (Fig. 3E and F). Notably, this decrease was observed only in HEK293T cells transfected with the SOD2-WT plasmid, while the reduction was less marked in those transfected with the SOD2-K68R mutant plasmid (Fig. 3G and H). These results indicate that β-OHB-induced Kbhb at K68 site enhances SOD2 stability by inhibiting its ubiquitin-dependent degradation.

### 3.4 The Kbhb modification at SOD2 K68 site mediates the inhibitory effects of β-OHB on NLRP3 inflammasome activation in macrophages

**Fig. 4:**
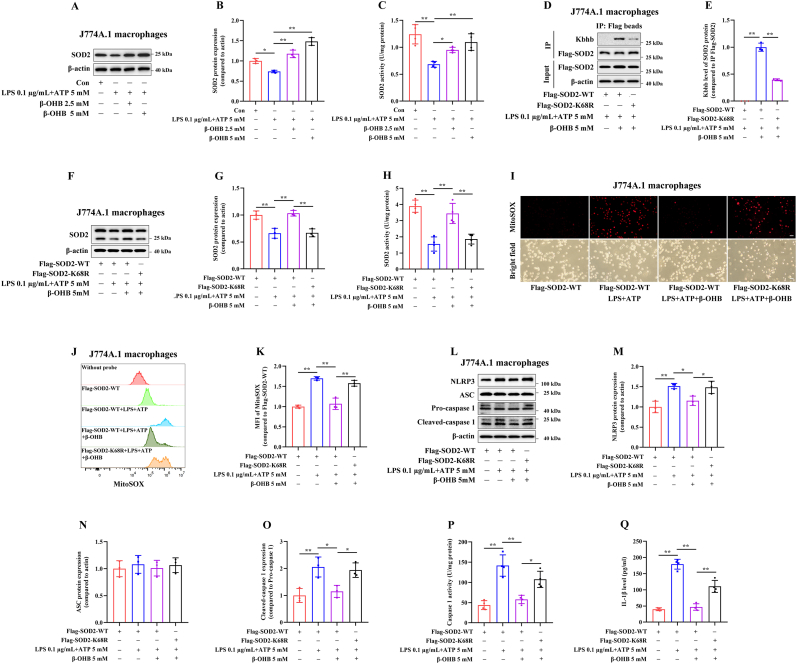
The Kbhb modification at the SOD2 K68 site mediates the inhibitory effects of β-OHB on NLRP3 inflammasome activation in macrophages. J774A.1 macrophages were treated with 2.5 or 5 mM β-OHB for 24 h, followed by incubation with 0.1 μg/mL LPS for 3 h and 5 mM ATP for 2 h. (A,B) Immunoblotting analysis of the SOD2 protein expression (n = 3). (C) The intracellular enzymatic activity of SOD2 was measured (n = 4). J774A.1 macrophages were transfected with the indicated plasmids and treated with or without 5 mM β-OHB for 24 h, followed by incubation with 0.1 μg/mL LPS for 3 h and 5 mM ATP for 2 h. (D,E) immunoprecipitation using anti-Flag beads and immunoblotting analysis of SOD2 Kbhb levels (n = 3). (F,G) Immunoblotting analysis of the SOD2 protein expression (n = 3). (H) Measurement of the intracellular enzymatic activity of SOD2 (n = 4). (I) Representative confocal microscope images of MitoSOX staining, Scale bar = 25 μm. (J,K) Representative flow cytometry images of MitoSOX staining. The histogram represents the mitochondrial ROS levels (n = 3). **(L**–**O)** Immunoblotting analysis of the protein expression of NLRP3, ASC and cleaved-caspase 1 (n = 3). (P) Measurement of the intracellular caspase-1 activity by colorimetry. (Q) The IL-1β levels in the supernatant measured by ELISA. ∗*p* < 0.05, ∗∗*p* < 0.01.

As shown in Fig. 4A–C, treatment with 2.5 or 5 mM β-OHB for 24 h significantly increased both the protein expression and enzymatic activity of SOD2 in J774A.1 macrophages stimulated with LPS + ATP. Treatment with β-OHB also obviously reduced mitochondrial ROS levels, as indicated by MitoSOX™ Red staining (SFig. 3A–C), and was accompanied by decreased protein levels of NLRP3 and cleaved caspase-1 (SFig. 3D–G). In addition, β-OHB lowered intracellular caspase-1 activity (SFig. 3H) and reduced IL-1β concentrations in the supernatant (SFig. 3I).

Transfection with the SOD2-K68R mutant plasmid reduced the β-OHB-induced SOD2 Kbhb levels in J774A.1 macrophages compared with transfection with the SOD2-WT plasmid (Fig. 4D and E). β-OHB treatment markedly increased both the protein expression and enzymatic activity of SOD2 in LPS + ATP-stimulated J774A.1 macrophages transfected with the SOD2-WT plasmid. Notably, the SOD2-K68R mutant led to a significant reduction in SOD2 protein levels (Fig. 4F and G) and enzymatic activity (Fig. 4H). In addition, the SOD2-K68R mutant evidently elevated mitochondrial ROS levels (Fig. 4I–K), which was accompanied by increased protein expression of NLRP3 and cleaved caspase-1 (Fig. 4L–O), enhanced intracellular caspase-1 activity (Fig. 4P), and higher IL-1β concentrations in the supernatant (Fig. 4Q). These results suggest that β-OHB-mediated Kbhb modification at SOD2 K68 site modulates NLRP3 inflammasome activation in macrophages.

### 3.5 The Kbhb modification at the SOD2 K68 site mediates the inhibitory effects of β-OHB on LPS-induced apoptosis in hepatocytes

**Fig. 5:**
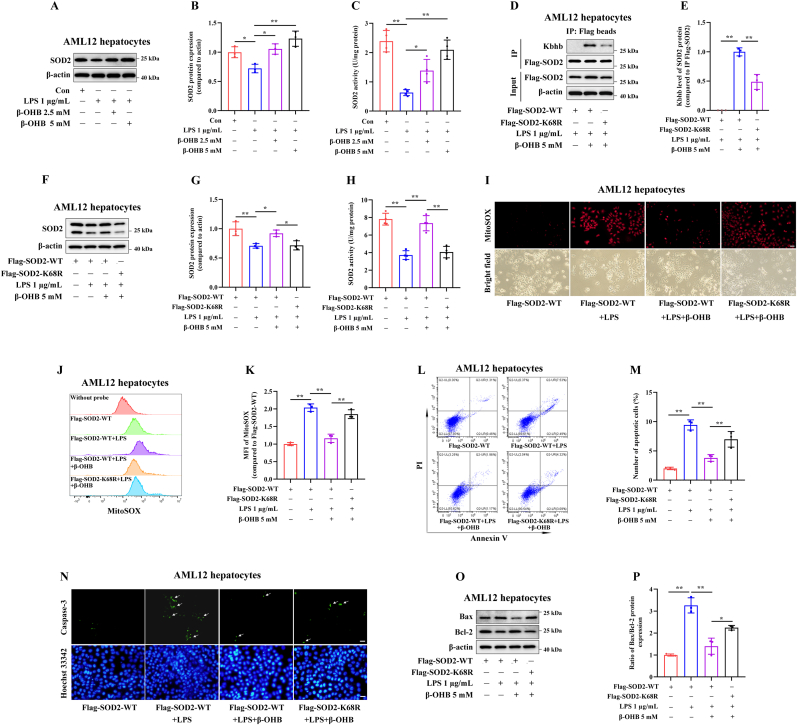
The Kbhb modification at the SOD2 K68 site mediates the inhibitory effects of β-OHB on LPS-induced apoptosis in hepatocytes. AML12 hepatocytes were treated with 2.5 or 5 mM β-OHB for 24 h, followed by incubation with 1 μg/mL LPS for 48 h. (A,B) Immunoblotting analysis of the SOD2 protein expression (n = 3). (C) The intracellular enzymatic activity of SOD2 was measured (n = 4). (A) AML12 hepatocytes were transfected with the indicated plasmids and treated with or without 5 mM β-OHB for 24 h, followed by incubation with 1 μg/mL LPS for 48 h. (D,E) immunoprecipitation using anti-Flag beads and immunoblotting analysis of SOD2 Kbhb levels (n = 3). (F,G) Immunoblotting analysis of the SOD2 protein expression (n = 3). (H) Measurement of the intracellular enzymatic activity of SOD2 (n = 4). (I) Representative confocal microscope images of MitoSOX staining, Scale bar = 25 μm. (J,K) Representative flow cytometry images of MitoSOX staining. The histogram represents the mitochondrial ROS levels (n = 3). **(L,M)** Representative flow cytometry images of Annexin V/PI staining. The histogram represents the percentage of apoptotic AML12 cells (n = 3). (N) Representative confocal microscope images of active caspase-3 staining, Scale bar = 50 μm. (O,P) Immunoblotting analysis of the protein expression of Bax and Bcl-2 (n = 3). ∗*p* < 0.05, ∗∗*p* < 0.01.

As shown in Fig. 5A–C, treatment with 2.5 or 5 mM β-OHB for 24 h significantly increased both the protein expression and enzymatic activity of SOD2 in AML12 hepatocytes stimulated with LPS. Treatment with β-OHB also notably reduced mitochondrial ROS levels, as indicated by MitoSOX™ Red staining (SFig. 4A–C), and decreased the percentage of apoptotic AML12 cells, as determined by Annexin V/PI staining (SFig. 4D and E). Additionally, β-OHB reduced the active caspase-3 expression (SFig. 4F) and lowered the Bax/Bcl-2 ratio, a pro-apoptotic marker (SFig. 4G and H).

Transfection with the SOD2-K68R mutant plasmid reduced β-OHB-induced SOD2 Kbhb levels in AML12 hepatocytes compared with transfection with the SOD2-WT plasmid (Fig. 5D and E). β-OHB treatment markedly increased both the protein expression and enzymatic activity of SOD2 in LPS-stimulated AML12 hepatocytes transfected with the SOD2-WT plasmid. Notably, the SOD2-K68R mutant significantly reduced SOD2 protein levels (Fig. 5F and G) and enzymatic activity (Fig. 5H). In addition, the SOD2-K68R mutant evidently elevated mitochondrial ROS levels (Fig. 5I–K), which was accompanied by an increased percentage of apoptotic AML12 cells (Fig. 5L and M), enhanced active caspase-3 expression (Fig. 5N), and a higher Bax/Bcl-2 ratio (Fig. 5O and P). These findings suggest that the Kbhb modification at the SOD2 K68 site, mediated by β-OHB, modulates LPS-induced apoptosis in hepatocytes.

### 3.6 KE administration improves liver injury in LPS-induced septic mice

**Fig. 6:**
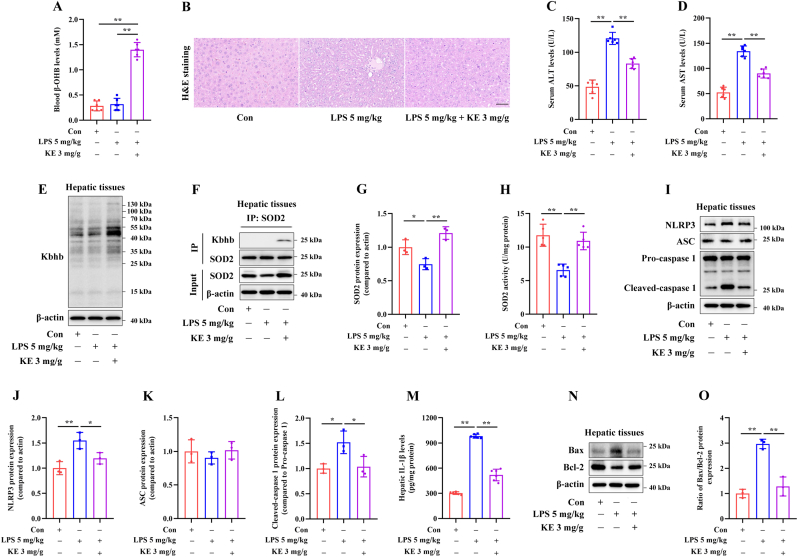
KE administration improves liver injury in LPS-induced septic mice. Nine-week-old male C57BL/6 mice were orally administered 3 mg/g KE per day for 3 days, followed by intraperitoneal injection of 5 mg/kg LPS for 24 h. (A) The circulating β-OHB levels were measured (n = 6). (B) The liver sections of mice from each group were stained with H&E, Scale bar = 50 μm, (n = 3). (C,D) Measurement of the serum ALT and AST levels by colorimetry (n = 6). (E) Immunoblotting analysis of the Kbhb modification in the hepatic tissues (n = 3). (F,G) After immunoprecipitation using anti-SOD2 antibodies, immunoblotting analysis of the SOD2 protein expression and its Kbhb levels in hepatic tissues was performed (n = 3). (H) Measurement of the hepatic enzymatic activity of SOD2 (n = 5). **(I**–**L)** Immunoblotting analysis of the hepatic NLRP3, ASC and cleaved-caspase 1 protein expression (n = 3). (M) The IL-1β levels in the hepatic tissues measured by ELISA (n = 6). (N,O) Immunoblotting analysis of Bax and Bcl-2 protein expression in the hepatic tissues (n = 3). ∗*p* < 0.05, ∗∗*p* < 0.01.

Three-day KE administration significantly increased circulating β-OHB levels in mice (Fig. 6A). As shown in Fig. 6B, compared with the Con mice, LPS injection induced evident pathological changes, including disordered arrangement of hepatocytes, ballooning degeneration, inflammatory cells infiltration, and spotty necrosis. KE treatment resulted in reduced hepatocyte swelling and milder inflammation in liver tissues. KE administration led to a significant decrease in serum ALT and AST levels in LPS-injected mice (Fig. 6C and D).

Furthermore, KE treatment markedly increased the overall Kbhb modification and SOD2 Kbhb levels (Fig. 6E and F), and enhanced both the protein expression and enzymatic activity of SOD2 (Fig. 6F–H) in liver tissues from LPS-injected mice. Additionally, KE administration significantly reduced the hepatic protein expression of NLRP3 and cleaved caspase-1 (Fig. 6I–L), decreased hepatic IL-1β levels (Fig. 6M), and increased the Bax/Bcl-2 ratio (Fig. 6N and O).

### 3.7 The SOD2-K68R mutant attenuates the protective effects of KE on LPS-induced septic liver injury in mice

**Fig. 7:**
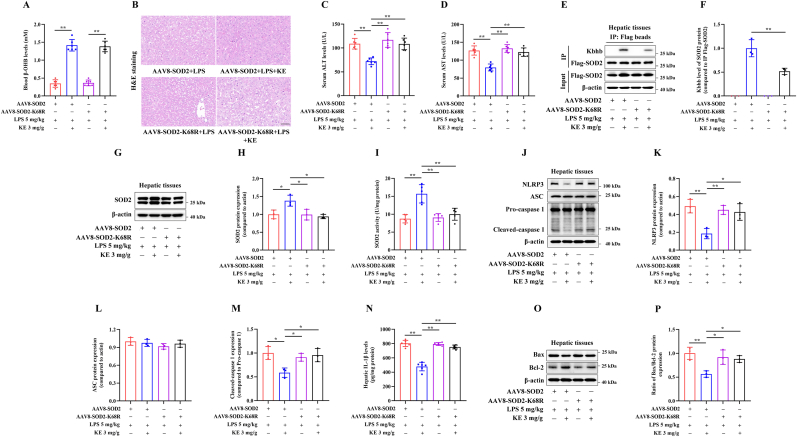
The SOD2-K68R mutant attenuates the protective effects of KE on LPS-induced septic liver injury in mice. Nine-week-old male C57BL/6 mice were transduced with AAV8-SOD2 or AAV8-SOD2-K68R vectors via tail vein injection. After 2 weeks of injection, the mice were subjected to KE + LPS treatment. (A) The circulating β-OHB levels were measured (n = 6). (B) The liver sections of mice from each group were stained with H&E, Scale bar = 50 μm, (n = 3). (C,D) Measurement of the serum ALT and AST levels by colorimetry (n = 6). (E,F) After immunoprecipitation using anti-Flag beads, immunoblotting analysis of the Flag-tagged protein and its Kbhb levels in hepatic tissues was performed (n = 3). (G,H) Immunoblotting analysis of the SOD2 protein expression in the hepatic tissues (n = 3). (I) Measurement of the hepatic enzymatic activity of SOD2 (n = 5). **(J**–**M)** Immunoblotting analysis of the hepatic NLRP3, ASC and cleaved-caspase 1 protein expression (n = 3). (N) The IL-1β levels in the hepatic tissues measured by ELISA (n = 6). (O,P) Immunoblotting analysis of Bax and Bcl-2 protein expression in the hepatic tissues (n = 3). ∗*p* < 0.05, ∗∗*p* < 0.01.

To investigate the effects of Kbhb modification at the SOD2 K68 site *in vivo*, we transduced C57BL/6 mice with AAV8-SOD2 or AAV8-SOD2-K68R vectors via tail vein injection. KE administration significantly increased circulating β-OHB levels in mice (Fig. 7A). Treatment with KE significantly improved liver pathological damage and reduced ALT and AST levels in LPS-injected mice transduced with the AAV8-SOD2 vector (Fig. 7B–D). However, compared with AAV8-SOD2 vector, transduction with AAV8-SOD2-K68R vector inhibited the protective effects of KE on liver damage in LPS-injected mice (Fig. 7B–D).

As shown in Fig. 7E–I, compared with transduction using the AAV8-SOD2 vector, transduction with the AAV8-SOD2-K68R vector reduced KE-induced hepatic SOD2 Kbhb levels. Additionally, it diminished the KE treatment-induced upregulation of SOD2 protein expression and enzymatic activity in LPS-injected mice (Fig. 7E–I). Furthermore, transduction with the AAV8-SOD2-K68R vector attenuated the inhibitory effects of KE on the protein expression of NLRP3 and cleaved caspase-1 (Fig. 7J–M), hepatic IL-1β production (Fig. 7N), and the Bax/Bcl-2 ratio (Fig. 7O and P).

## 4 Discussion

Liver injury is a common and early manifestation of sepsis, significantly contributing to the increased risk of hospitalisation and mortality associated with the condition [2,3]. Hepatocytes are central to liver function, performing metabolism, detoxification and protein synthesis. Irreversible loss of function in hepatocytes leads to liver injury. Hepatic macrophages regulate immune responses and facilitate pathogen clearance; however, excessive pro-inflammatory activation of hepatic macrophages is a key contributor to septic liver injury [29]. Furthermore, oxidative stress plays a pivotal role in the pathogenesis of liver damage during sepsis [[10], [11], [12], [13], [14]]. Thus, targeting oxidative stress may represent a promising therapeutic strategy for septic liver injury [30].

In this study, we investigated the role of β-OHB, a major component of ketone bodies, in modulating oxidative stress-induced septic liver injury through a novel PTM known as Kbhb. We focused on SOD2, a key mitochondrial antioxidant enzyme that is essential for maintaining mitochondrial homeostasis and protecting cells from oxidative damage [31]. Specifically, we demonstrated that β-OHB regulates the expression and activity of SOD2 via Kbhb modification in both hepatocytes and macrophages.

We identified lysine 68 (K68) of SOD2 as the primary site of Kbhb modification. This β-OHB–induced modification enhances SOD2 protein stability by inhibiting the ubiquitin–proteasome pathway, thereby increasing its expression and enzymatic activity and reducing mitochondrial ROS production. This conclusion is further supported by our findings showing that mutation of lysine 68 to arginine (SOD2 K68R mutant), which blocks Kbhb modification, significantly abolished these β-OHB–mediated effects.

Notably, K68 is located near the active site of SOD2 and is known to be susceptible to other PTMs, such as acetylation and succinylation, both of which impair its enzymatic activity [[32], [33], [34]]. In contrast, our data suggest that β-OHB-induced Kbhb at this site enhances both the expression and function of SOD2. These results are consistent with previous studies showing that β-OHB-induced Kbhb stabilizes proteins such as Snail and SLC25A5 [22,26]. Collectively, these findings indicate that different PTMs can exert distinct, even opposing, effects on SOD2 function. Future research should explore the dynamic interplay between Kbhb and other PTMs, which may finely regulate SOD2 activity under various physiological and pathological conditions.

During sepsis, excessive production of mitochondrial ROS disrupts hepatocyte mitochondrial function, leading to the activation of pro-apoptotic signalling pathways, including Bax/Bcl-2 imbalance, cytochrome *c* release and caspase activation, ultimately leading to apoptosis [35,36]. Concurrently, in hepatic macrophages, mitochondrial ROS act as upstream signals that trigger the activation of the NLRP3 inflammasome, resulting in caspase-1 cleavage and the maturation and release of pro-inflammatory cytokines such as IL-1β, thereby amplifying systemic inflammation [37,38].

By stabilizing SOD2 and enhancing its expression and activity through Kbhb modification, β-OHB effectively reduces mitochondrial ROS levels. This, in turn, suppresses NLRP3 inflammasome activation in J774A.1 macrophages and inhibits apoptosis in AML12 hepatocytes. Moreover, administration of KE, an exogenous compound that elevates β-OHB levels, significantly attenuates sepsis-induced liver injury *in vivo*. The protective effects of β-OHB were markedly reduced in both *in vitro* and *in vivo* models expressing the SOD2-K68R mutant, indicating that the β-OHB–SOD2–Kbhb axis is crucial for the therapeutic efficacy of β-OHB in septic liver injury. Future studies should investigate whether this pathway is also involved in other sepsis-related organ injuries, such as septic cardiomyopathy, acute lung injury or acute kidney injury, which are driven by oxidative stress and inflammasome activation as well.

This study has several limitations. First, potential upstream regulators of SOD2 protein Kbhb modification, such as whether β-OHB regulates sirtuin 3 levels to influence SOD2 Kbhb modification, require further investigation, especially given that sirtuin 3 has been shown to selectively remove Kbhb modification from histones [39]. Second, it is important to recognize that, in addition to Kbhb modification, β-OHB may also contribute to the upregulation of SOD2 mRNA transcription through the forkhead box O3 and nuclear factor-erythroid 2-related factor-2 pathways [15,40], indicating that the regulatory effects of β-OHB on SOD2 occur at multiple levels. Third, the increase in SOD2 activity following β-OHB/KE treatment was not proportional to the increase in SOD2 protein expression, which may be influenced by multiple regulatory mechanisms, including cofactor availability, the redox environment, and enzyme stability [41,42]; these mechanisms also warrant further investigation.

**Fig. 8:**
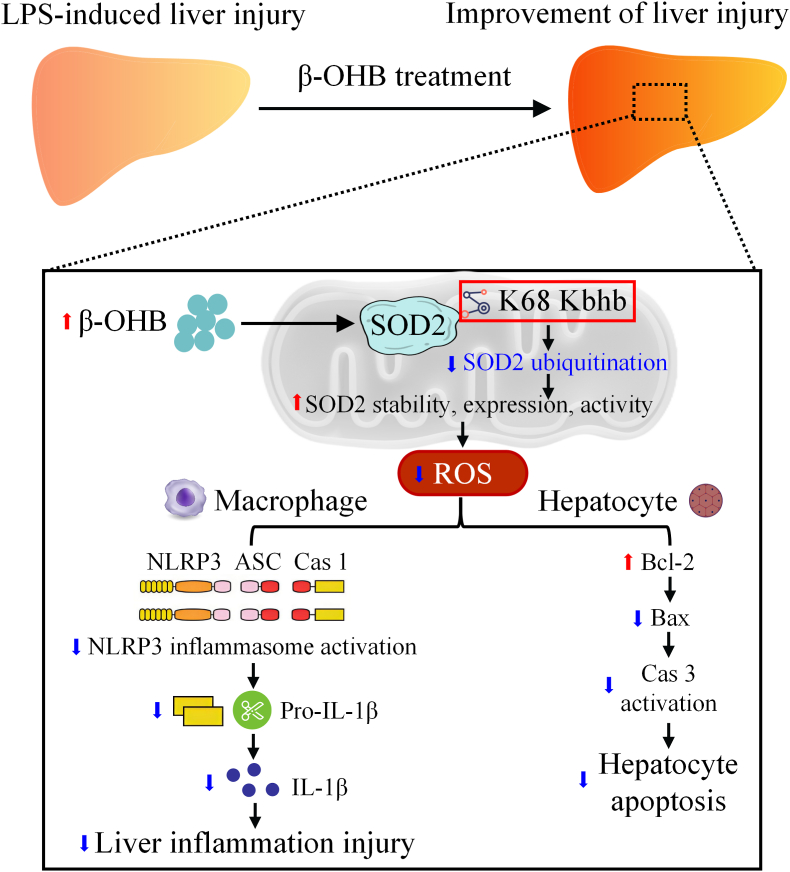
A regulatory model for the β-OHB-induced improvement of septic liver injury, mediated through SOD2 Kbhb modification.

In conclusion, this study is the first to demonstrate that β-OHB alleviates LPS-induced liver injury by inducing Kbhb modification at the K68 site of the antioxidant enzyme SOD2. This modification enhances the stability, expression and enzymatic activity of SOD2, thereby reducing mitochondrial ROS levels. Consequently, it inhibits NLRP3 inflammasome–mediated IL-1β production in macrophages and attenuates hepatocyte apoptosis (Fig. 8). These findings advance our understanding of the biological functions of β-OHB and suggest a promising therapeutic strategy for the treatment of septic liver injury.

## Data sharing statement

Data on Kbhb-modified proteomics in the oxidative stress and redox pathways are presented in Table S1. Additional Kbhb-modified proteomics data are available upon request from the corresponding author.

## CRediT authorship contribution statement

**Ya-Ping Bai:** Data curation, Formal analysis, Methodology, Writing – original draft, Writing – review & editing. **Yan Zhang:** Conceptualization, Writing – original draft, Writing – review & editing. **Zhi-Yuan Chen:** Methodology. **Kai Li:** Methodology. **De-Guo Wang:** Writing – original draft, Writing – review & editing. **Shu-Jun Wan:** Methodology. **Cui-Wei Zhang:** Methodology. **Yue Sun:** Methodology. **Zhi-Chao Li:** Writing – original draft, Writing – review & editing. **Kun Lv:** Conceptualization, Writing – review & editing. **Lei Zha:** Formal analysis, Methodology, Writing – original draft, Writing – review & editing. **Xiang Kong:** Conceptualization, Data curation, Funding acquisition, Investigation, Supervision, Writing – original draft, Writing – review & editing.

## Declaration of competing interest

The authors declare that they have no known competing financial interests or personal relationships that could have appeared to influence the work reported in this paper.
