# Supplementary material for: β-Hydroxybutyrate ameliorates lipopolysaccharide-induced liver injury through β-hydroxybutyrylation of the SOD2 protein in mice
Source: Redox Biol. 2025 Nov 27;88:103949. doi: 10.1016/j.redox.2025.103949 (PMC12704266; doi:10.1016/j.redox.2025.103949)
Supplement: Multimedia component 2 [file mmc2.docx]

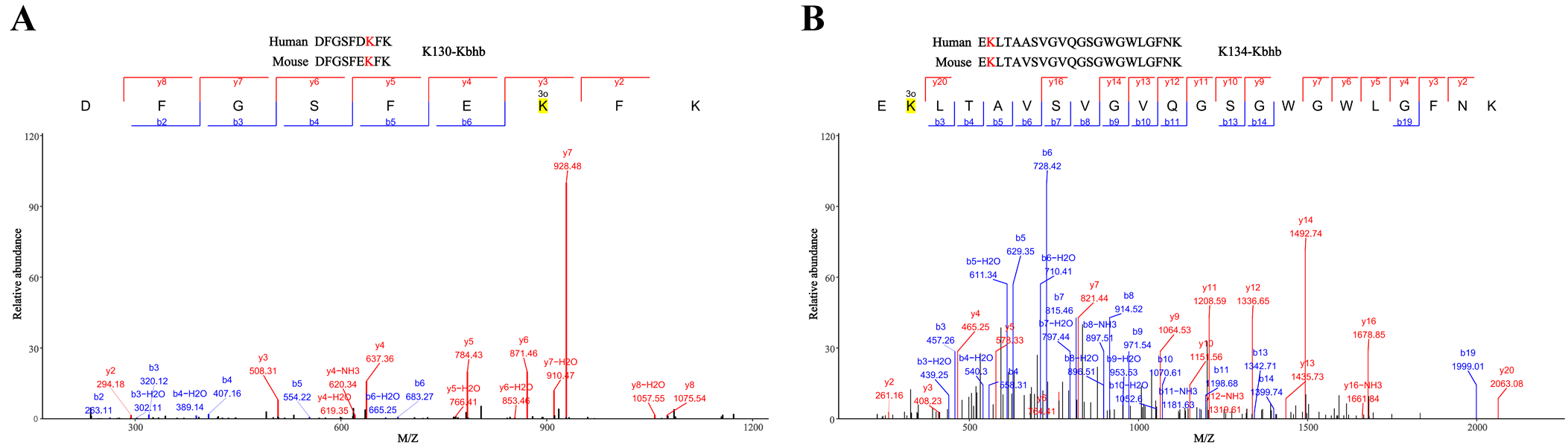


**Supplementary Fig. 1** (A,B) MS data showing SOD2 Kbhb modification sites (K130 and K134) identified in β-OHB-treated BMDMs. Top: conservation of Kbhb sites in human and mouse SOD2 proteins.


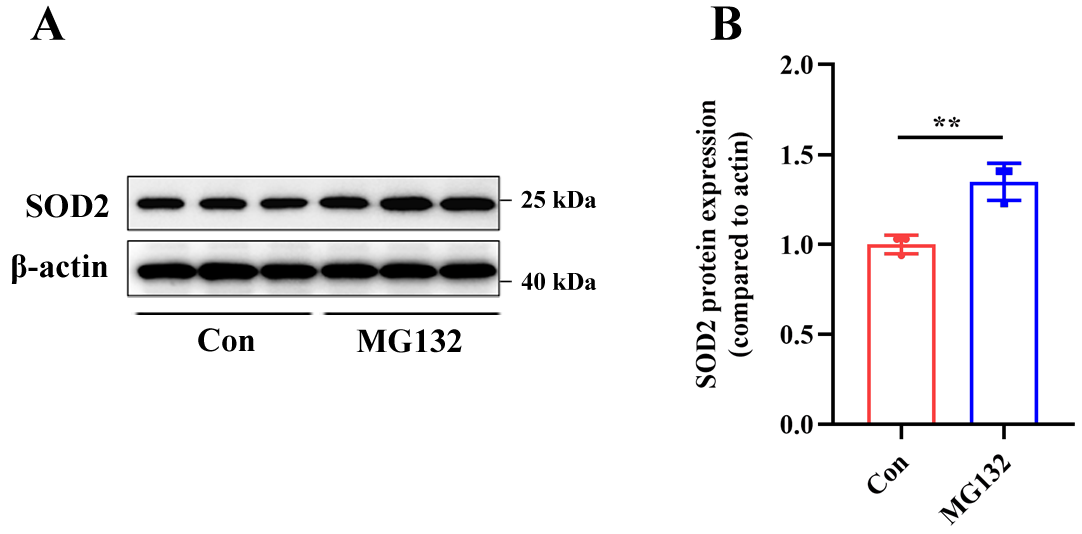


**Supplementary Fig. 2** (A,B) HEK293T cells were treated with or without 10 µM MG132 for 12 h. Immunoblotting analysis of the protein expression of SOD2 (n = 3).


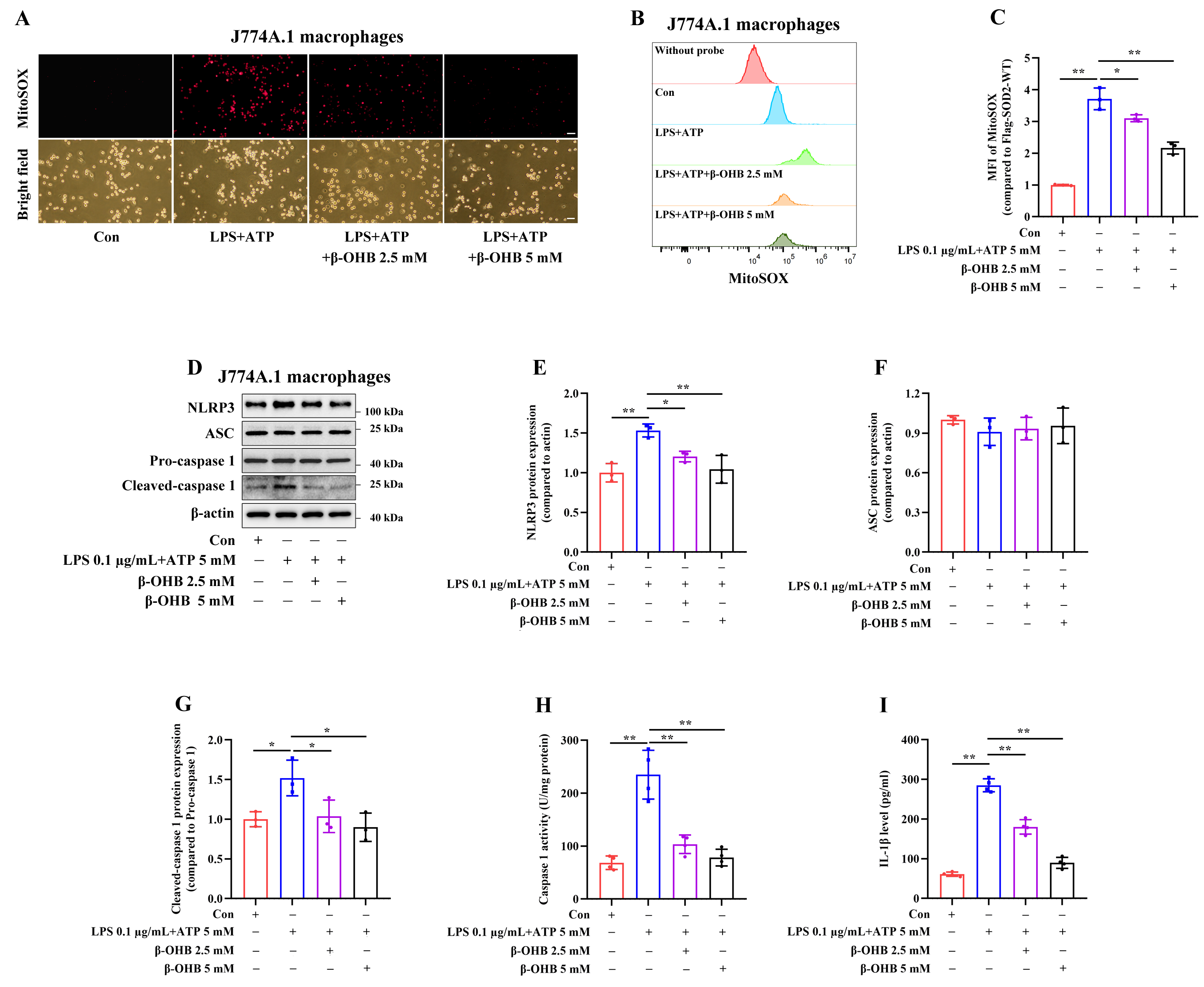


**Supplementary Fig. 3** J774A.1 macrophages were treated with 2.5 or 5 mM β-OHB for 24 h, followed by incubation with 0.1 µg/mL LPS for 3 h and 5 mM ATP for 2 h. (A) Representative confocal microscope images of MitoSOX staining, Scale bar = 25 μm. (B,C) Representative flow cytometry images of MitoSOX staining. The histogram represents the mitochondrial ROS levels (n = 3). (D–G) Immunoblotting analysis of the protein expression of NLRP3, ASC and cleaved-caspase 1 (n = 3). (H) The intracellular caspase-1 activity was measured using colorimetry. (I) The IL-1β levels in the supernatant were measured using ELISA. **p* < 0.05, ***p* < 0.01.


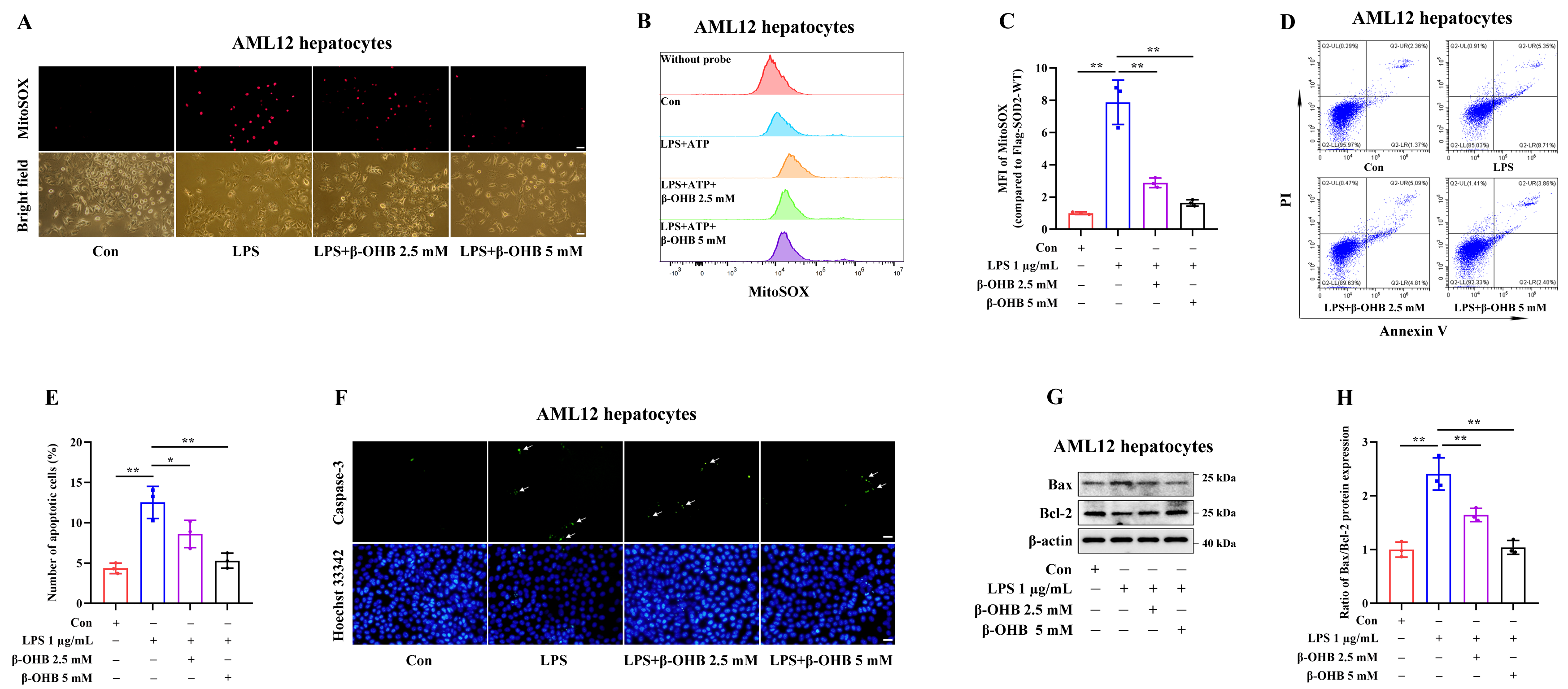


**Supplementary Fig. 4** AML12 hepatocytes were treated with 2.5 or 5 mM β-OHB for 24 h, followed by incubation with 1 µg/mL LPS for 48 h. (A) Representative confocal microscope images of MitoSOX staining, Scale bar = 25 μm. (B,C) Representative flow cytometry images of MitoSOX staining. The histogram represents the mitochondrial ROS levels (n = 3). (D,E) Representative flow cytometry images of Annexin V/PI staining. The histogram represents the percentage of apoptotic AML12 cells (n = 3). (F) Representative confocal microscope images of active caspase-3 staining, Scale bar = 50 μm. (G,H) Immunoblotting analysis of the protein expression of Bax and Bcl-2 (n = 3). **p* < 0.05, ***p* < 0.01.
