# Supplementary material for: β-Hydroxybutyrate ameliorates lipopolysaccharide-induced liver injury through β-hydroxybutyrylation of the SOD2 protein in mice
Source: Redox Biol. 2025 Nov 27;88:103949. doi: 10.1016/j.redox.2025.103949 (PMC12704266; doi:10.1016/j.redox.2025.103949)
Supplement: Multimedia component 3 [file mmc3.pdf]

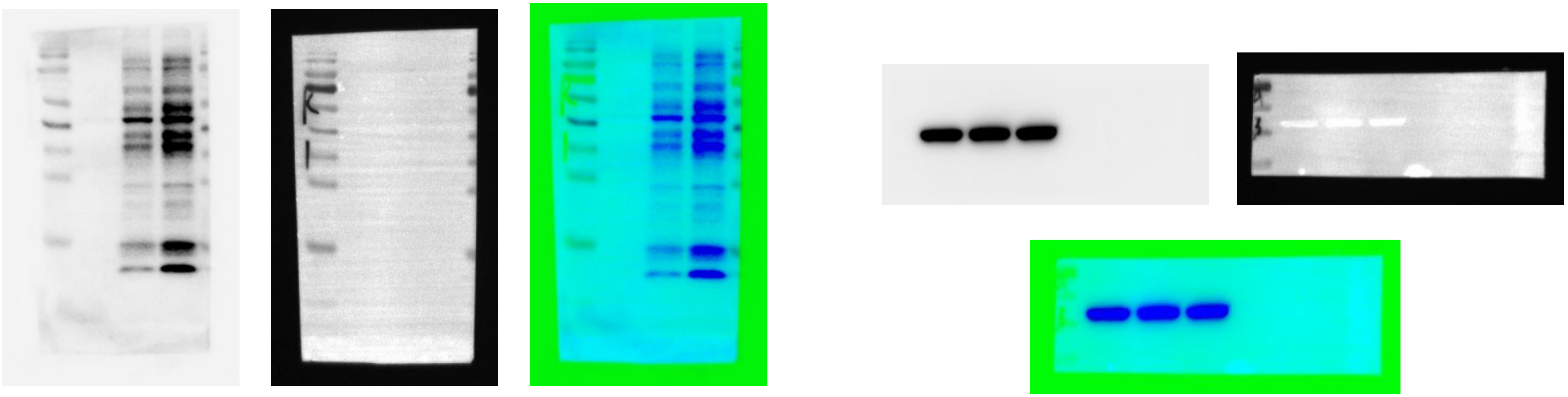

Kbhb β-actin

Fig.1C Immunoblotting analysis of the Kbhb modification in the J774A.1 cells

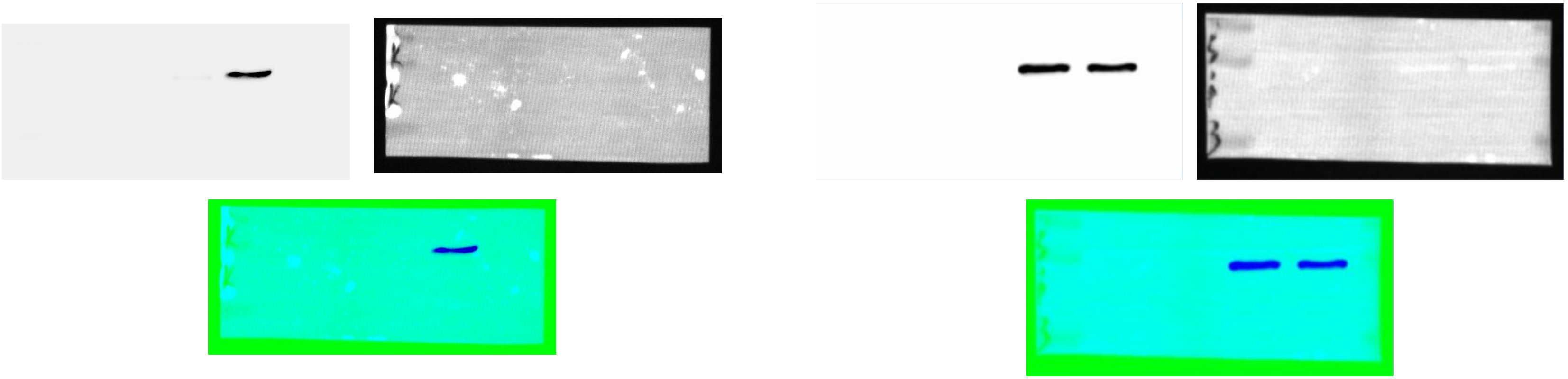

SOD2 antibody IP
WB analysis of Kbhb

SOD2 antibody IP
WB analysis of SO2

Fig.1D Co-IP analysis of the SOD2 Kbhb levels in J774A.1 cells

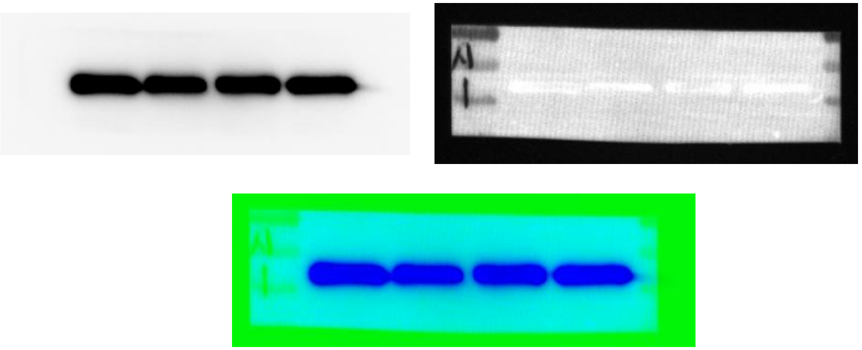

SOD2 antibody IP
WB analysis of β-actin in the whole-cell lysates (input)

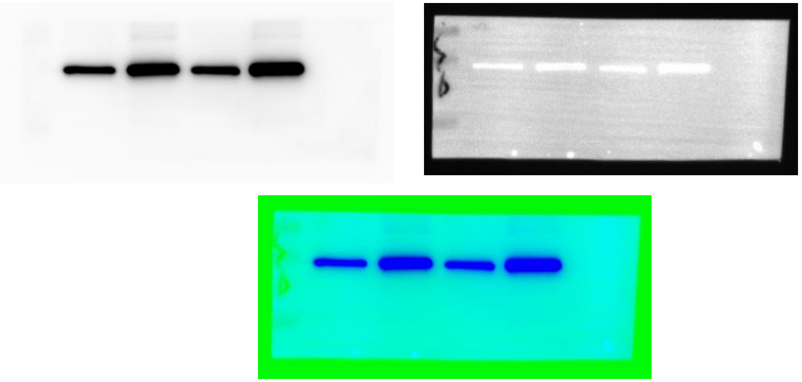

SOD2 antibody IP
WB analysis of SOD2 in the whole-cell lysates (input)

Fig.1D Co-IP analysis of the SOD2 Kbhb levels in J774A.1 cells

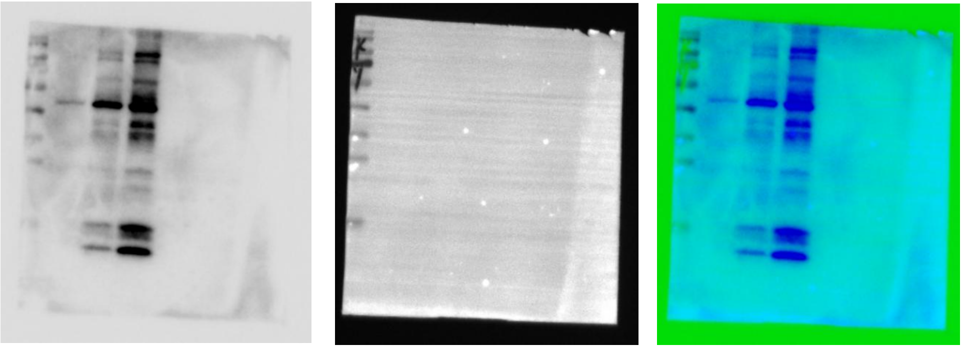

Kbhb

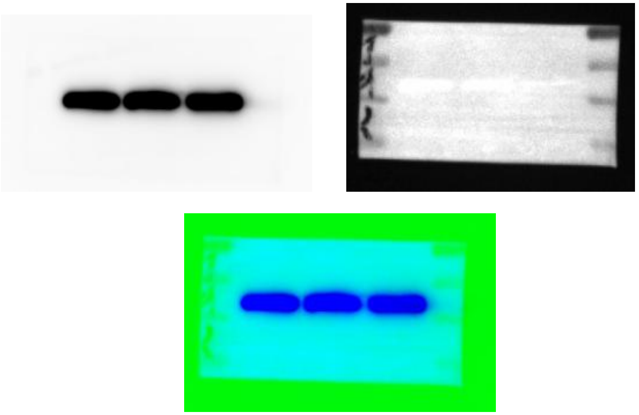

β-actin

Fig.1E Immunoblotting analysis of the Kbhb modification in the AML12 cells

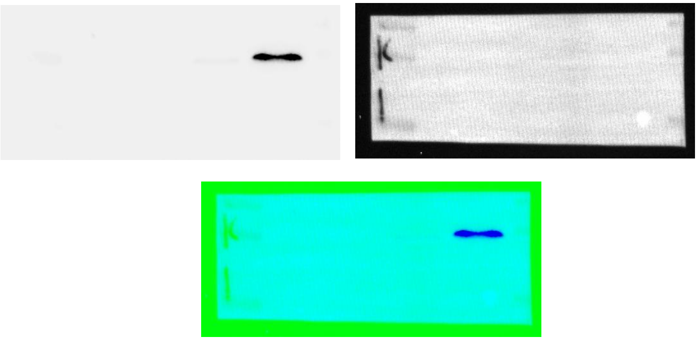

SOD2 antibody IP
WB analysis of Kbhb

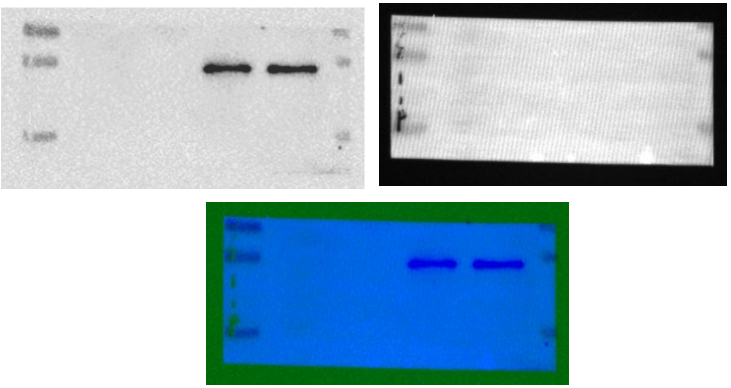

SOD2 antibody IP
WB analysis of SO2

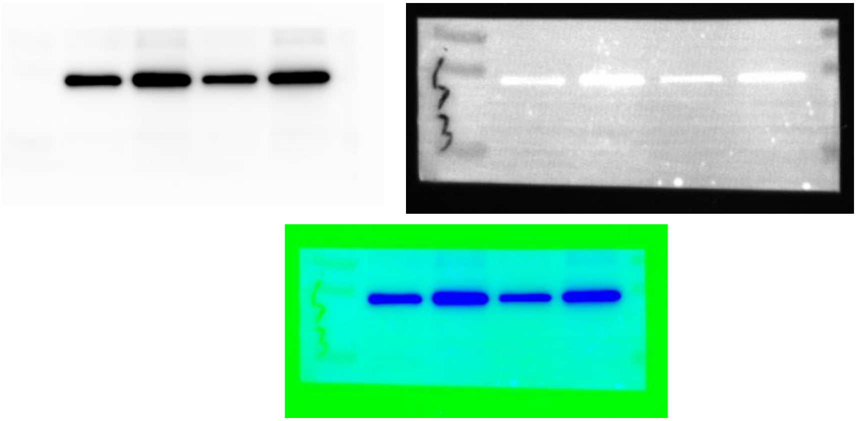

SOD2 antibody IP
WB analysis of SOD2 in the
whole-cell lysates (input)

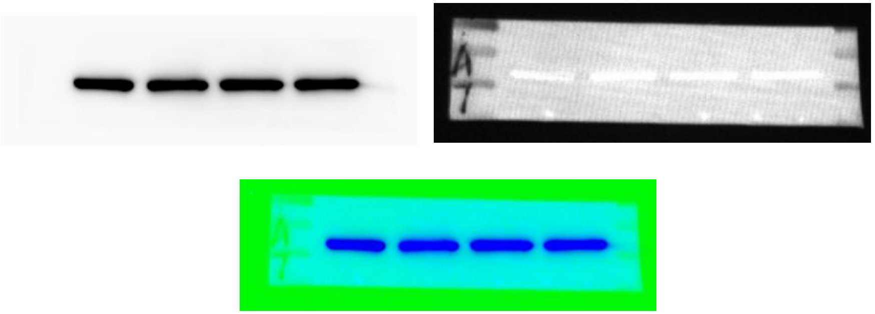

SOD2 antibody IP
WB analysis of β-actin in the
whole-cell lysates (input)

Fig.1F Co-IP analysis of the SOD2 Kbhb levels in AML12 cells

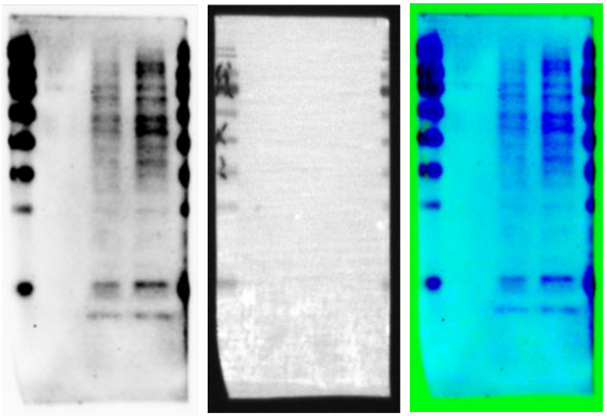

Kbhb

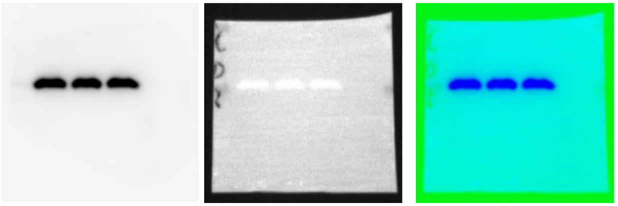

COX IV

Fig.1G Immunoblotting analysis of the Kbhb modification in the mitochondria of HEK293T cells

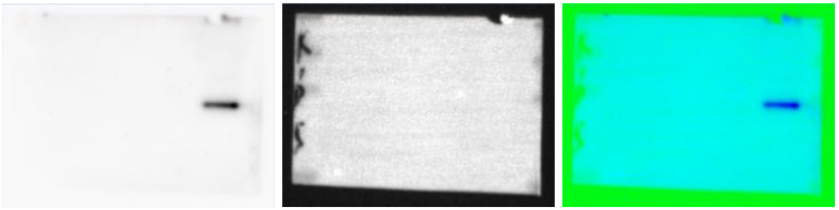

SOD2 antibody IP
WB analysis of Kbhb

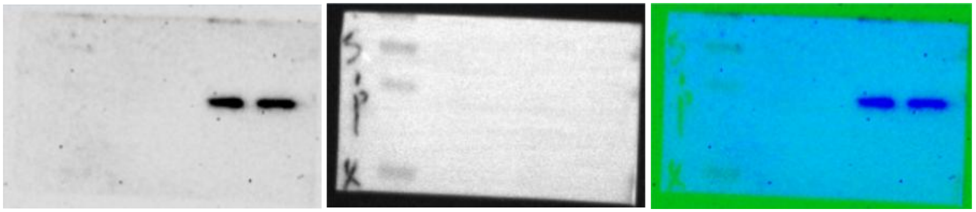

SOD2 antibody IP
WB analysis of SO2

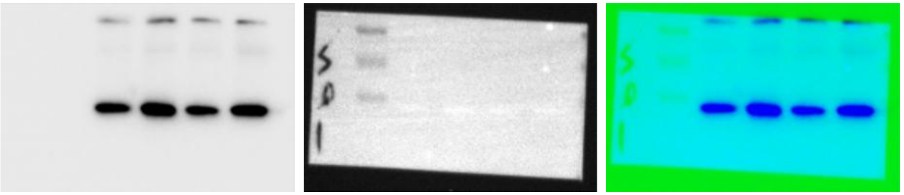

WB analysis of SOD2 in the mitochondria (input)

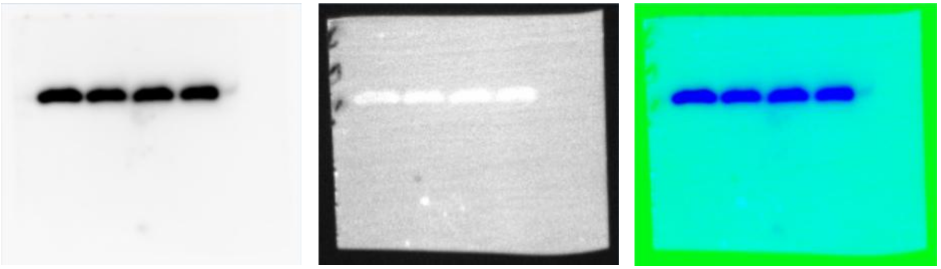

WB analysis of COX IV in the mitochondria (input)

Fig.1H Co-IP analysis of the SOD2 Kbhb levels in the mitochondria of HEK293T cells

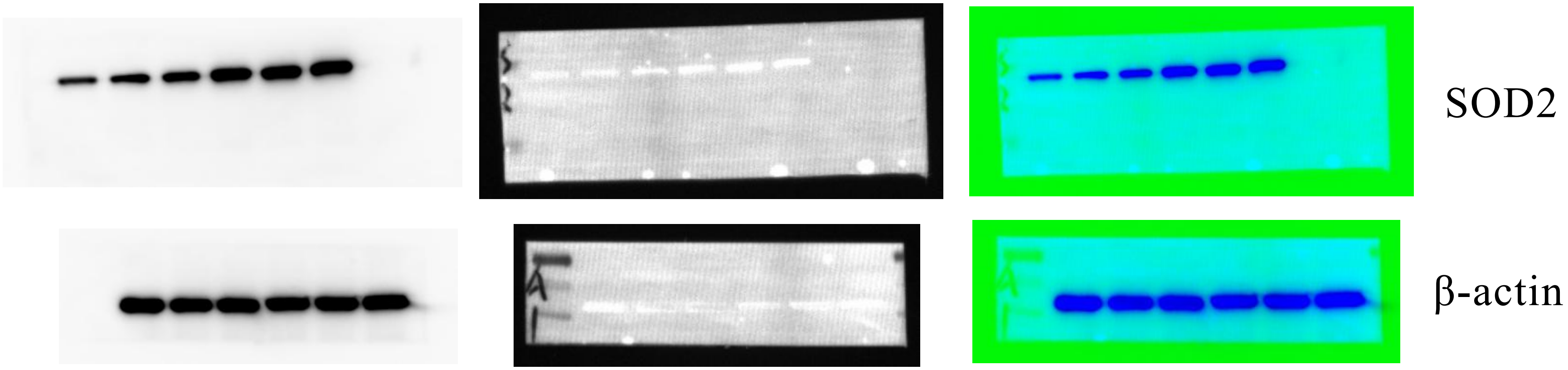

Fig.2A Immunoblotting analysis of the protein expression of SOD2

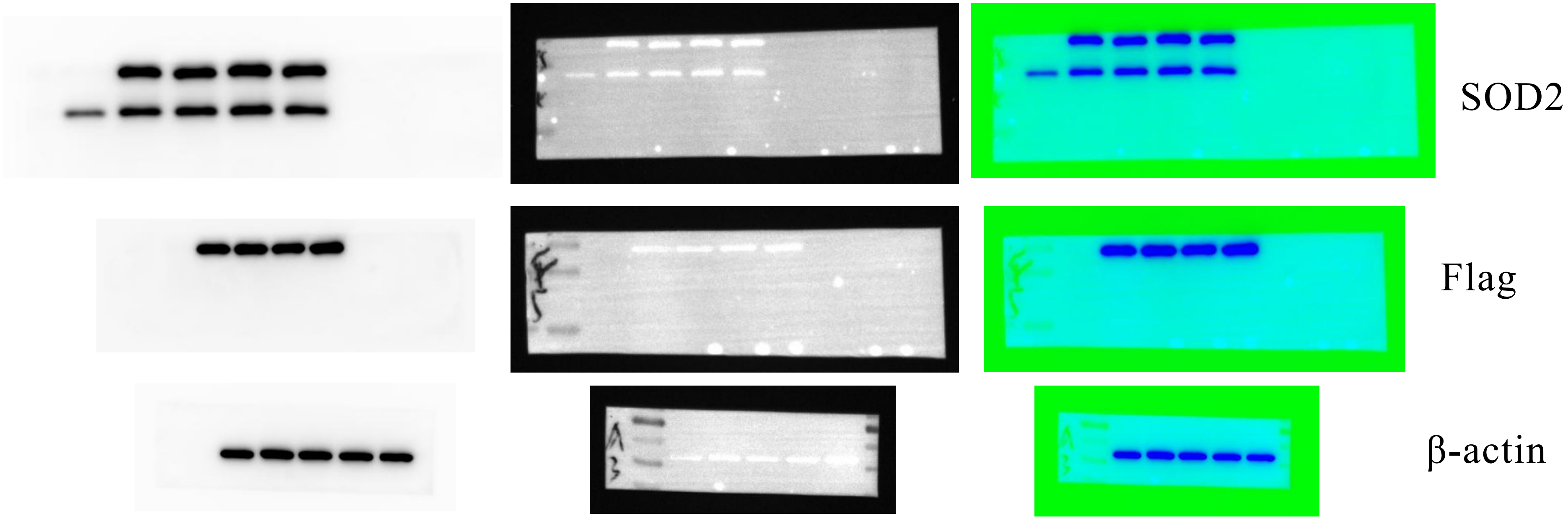

Fig.2G The overexpression efficiency of the Flag-SOD2-WT or -K to R mutant plasmids was assessed

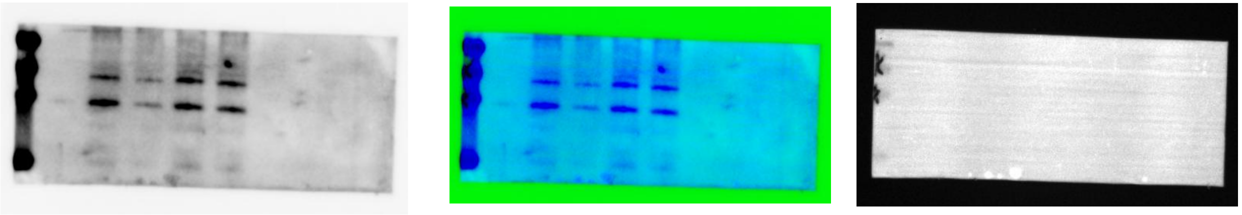

SOD2 antibody IP and WB analysis of Kbhb

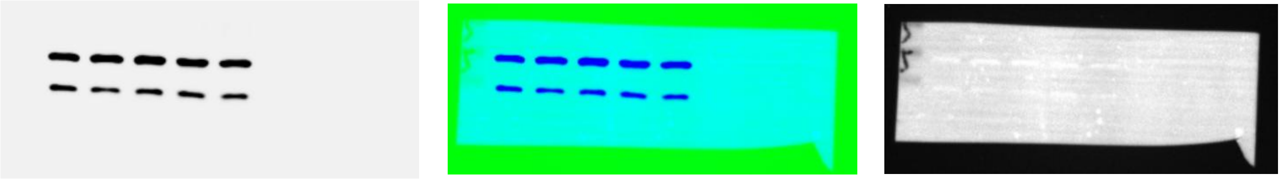

SOD2 antibody IP and WB analysis of SOD2

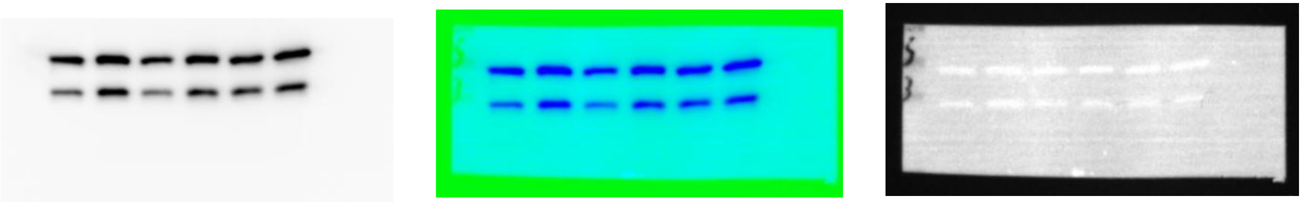

WB analysis of SOD2 in the whole-cell lysates (input)

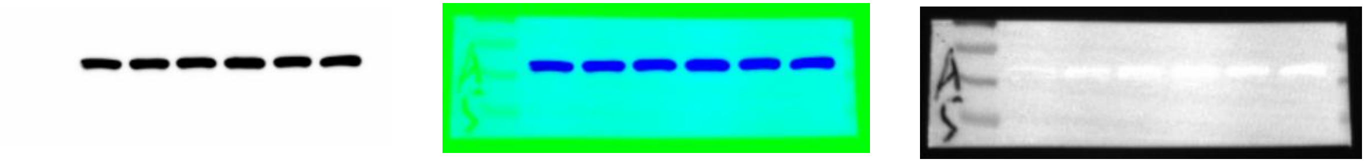

WB analysis of β-actin in the whole-cell lysates (input)

Fig.2H HEK293T cells were transfected with the indicated plasmids and treated with 5 mM β-OHB for 24 h, followed by IP using anti-SOD2 antibodies

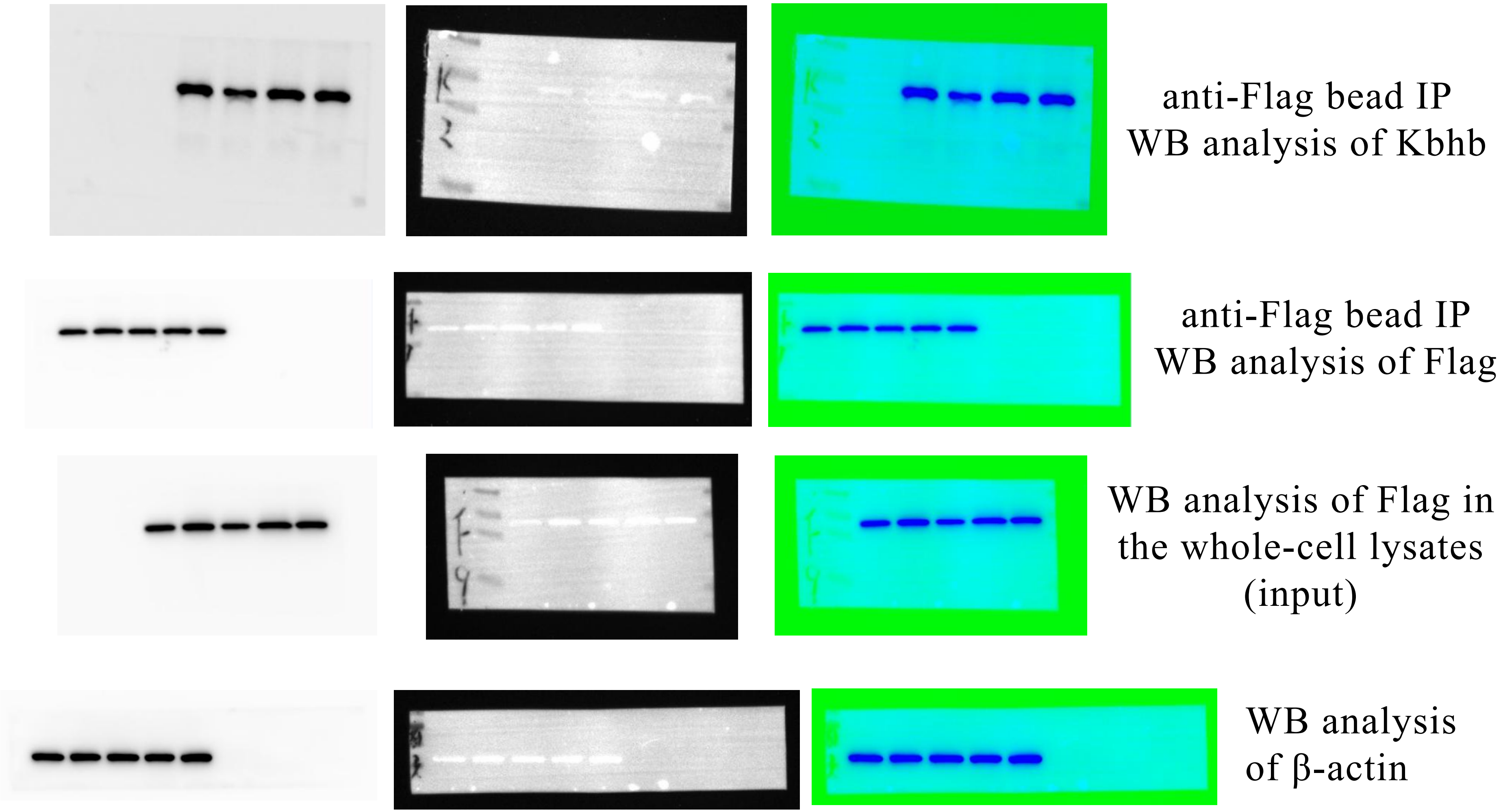

Fig.2I HEK293T cells were transfected with the indicated plasmids and treated with 5 mM β-OHB for 24 h, followed by IP using anti-Flag beads

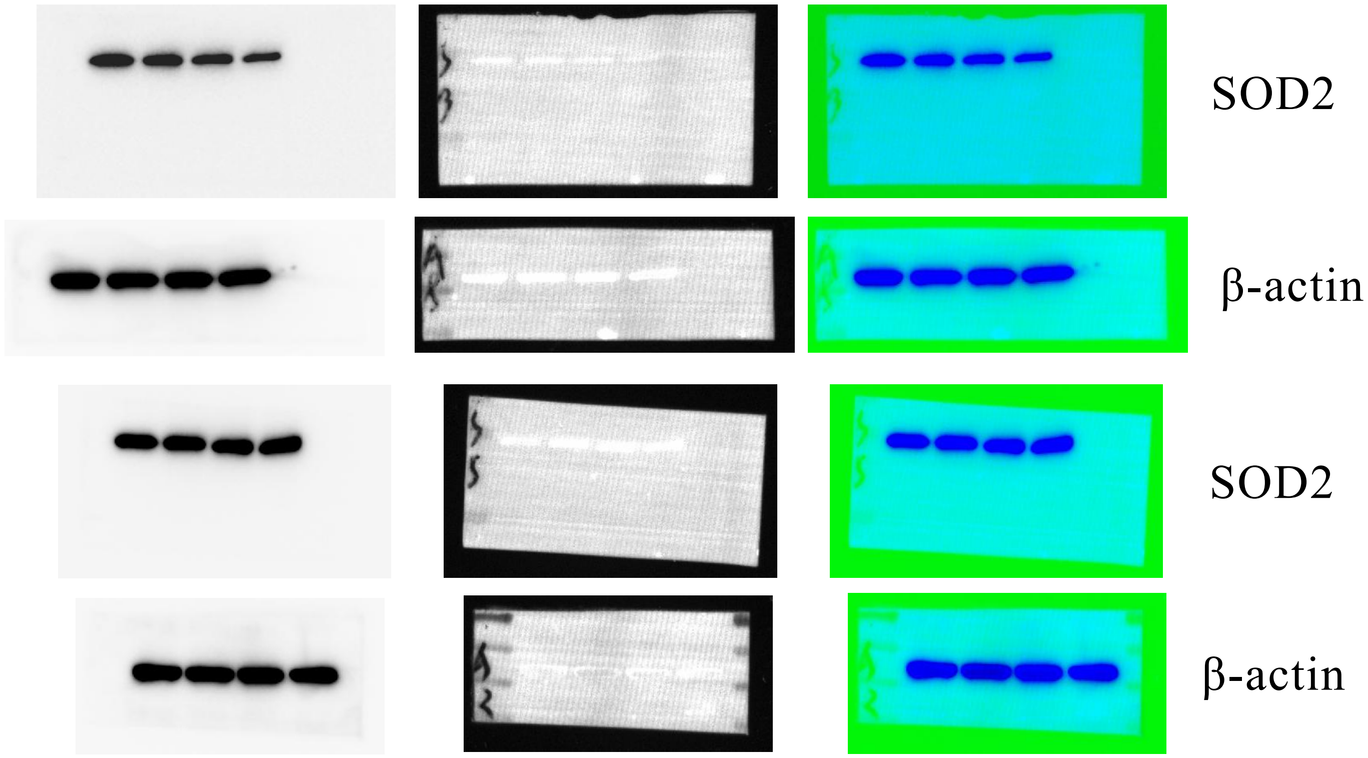

Fig.3A HEK293T cells were treated with or without 5 mM β-OHB for 24 h, followed by incubation with 100 μg/mL CHX for the indicated times. Immunoblotting analysis of the SOD2 protein expression

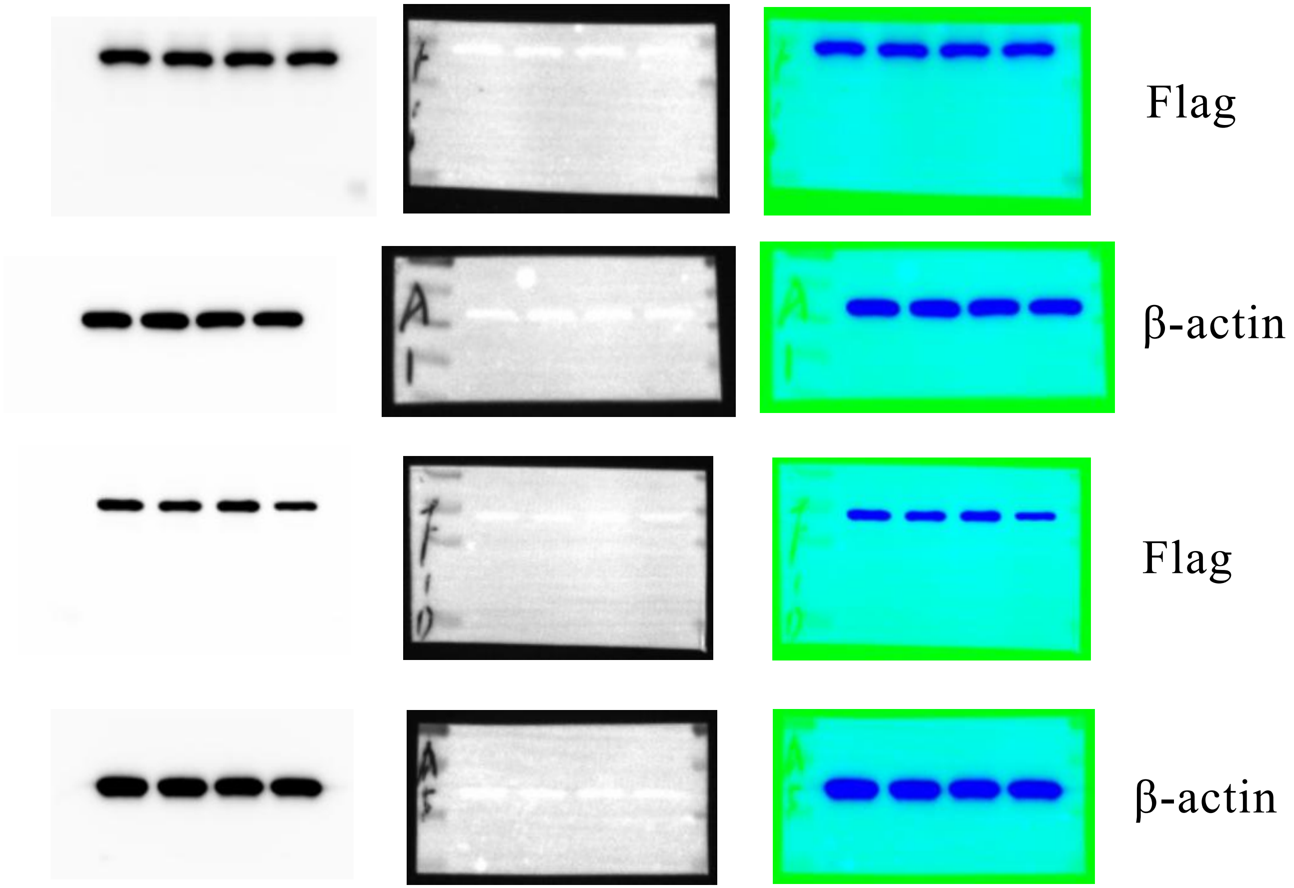

Fig.3C HEK293T cells were transfected with the indicated plasmids, treated with 5 mM β-OHB for 24 h, and then exposed to 100 μg/mL CHX for the indicated times. Immunoblotting analysis of the Flag-tagged protein expression

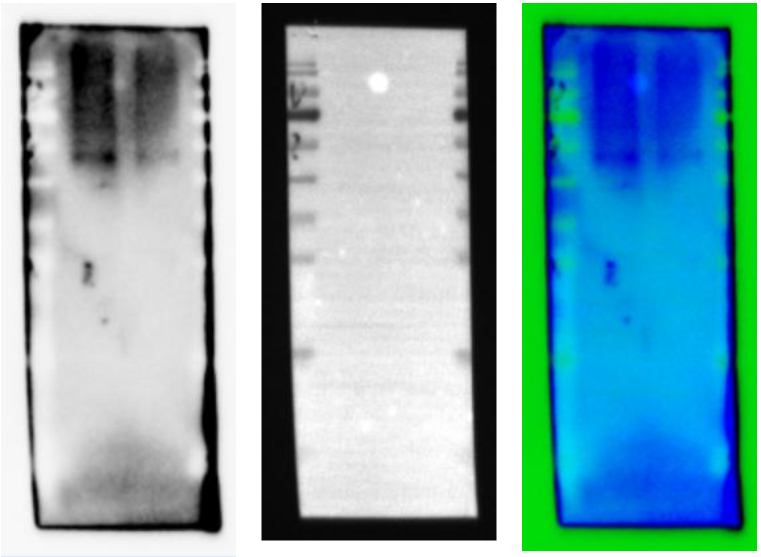

SOD2 antibody IP
WB analysis of ubiquitin

Fig.3E HEK293T cells were treated with or without 5 mM β-OHB for 24 h, then incubated with 10 μM MG132 for 12 h, followed by IP using anti-SOD2 antibodies

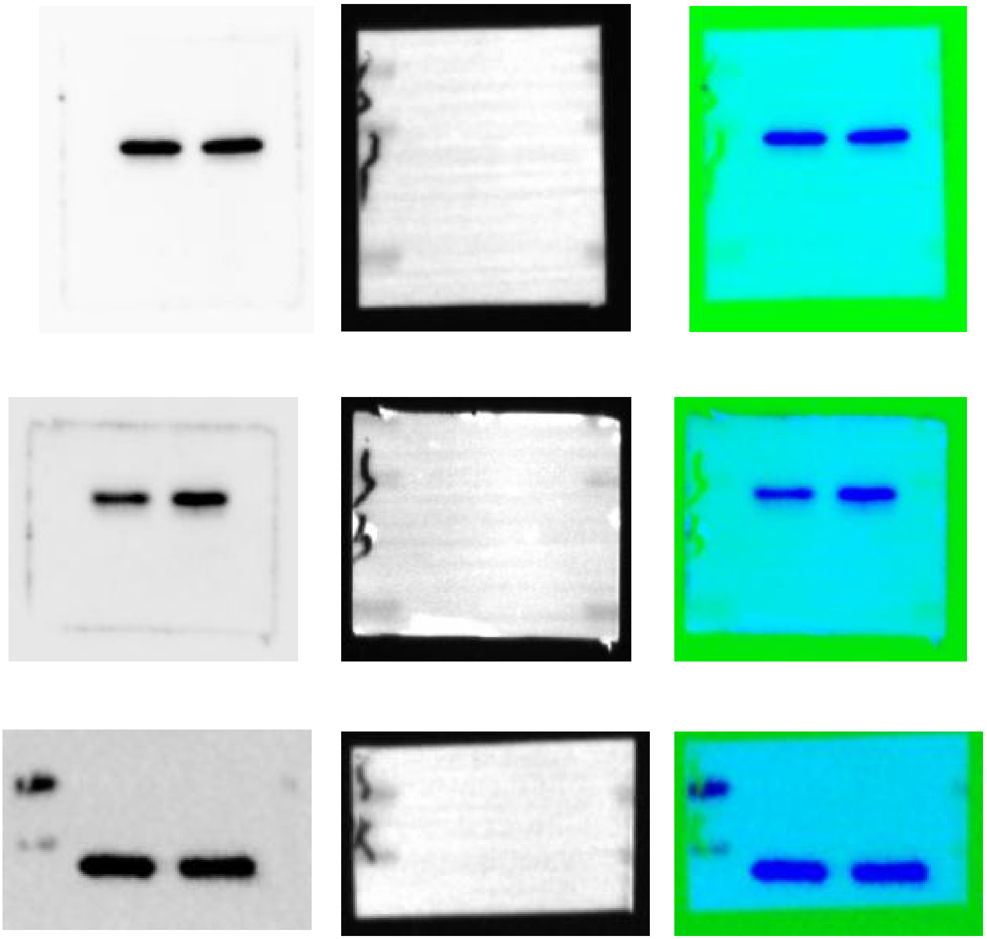

SOD2 antibody IP
WB analysis of SOD2

WB analysis of SOD2 in the whole-cell lysates (input)

WB analysis of β-actin in the whole-cell lysates (input)

Fig.3E HEK293T cells were treated with or without 5 mM β-OHB for 24 h, then incubated with 10 μM MG132 for 12 h, followed by IP using anti-SOD2 antibodies

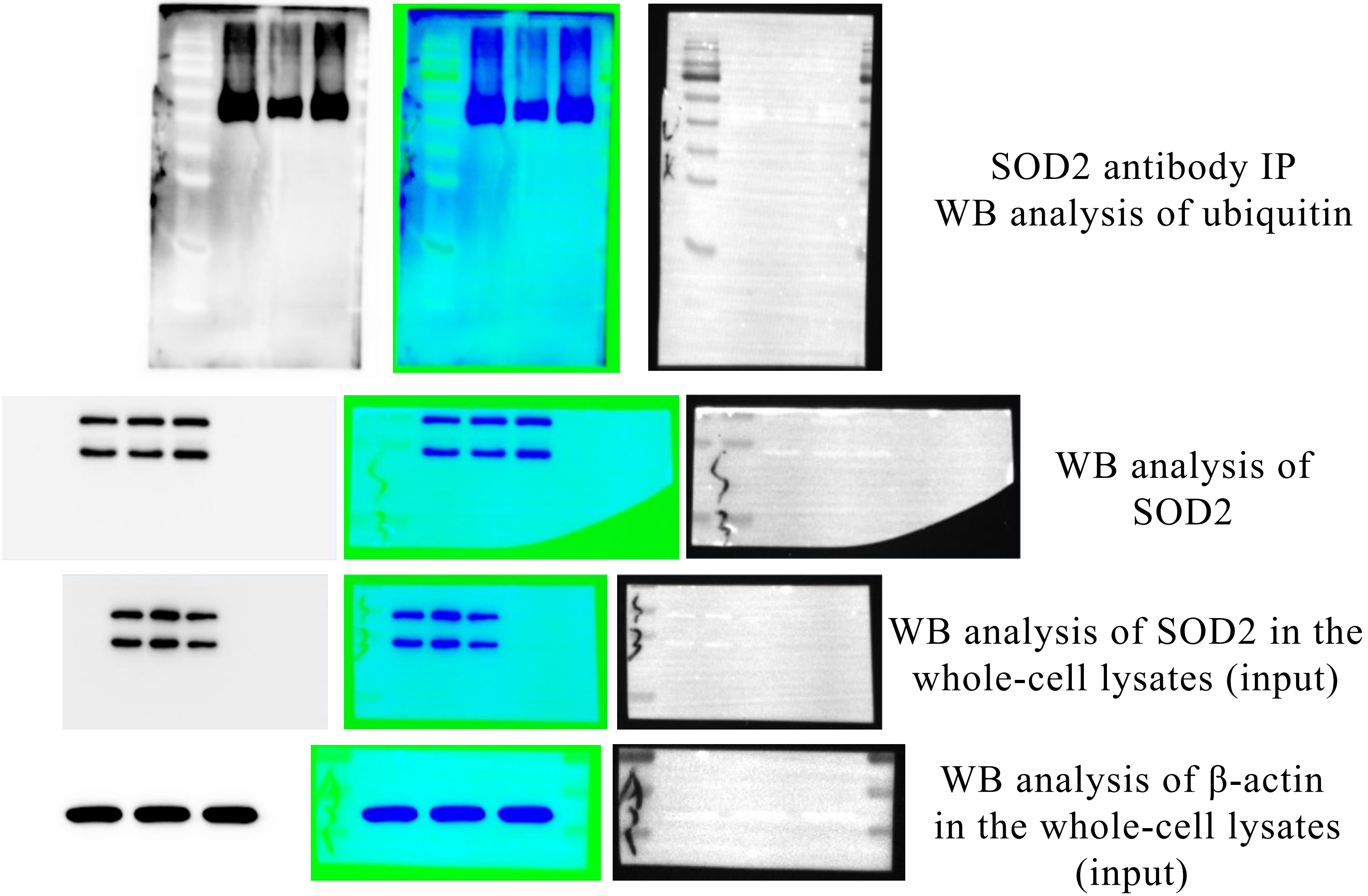

Fig.3G HEK293T cells were treated with or without 5 mM β-OHB for 24 h, then incubated with 10 μM MG132 for 12 h, followed by IP using anti-SOD2 antibodies

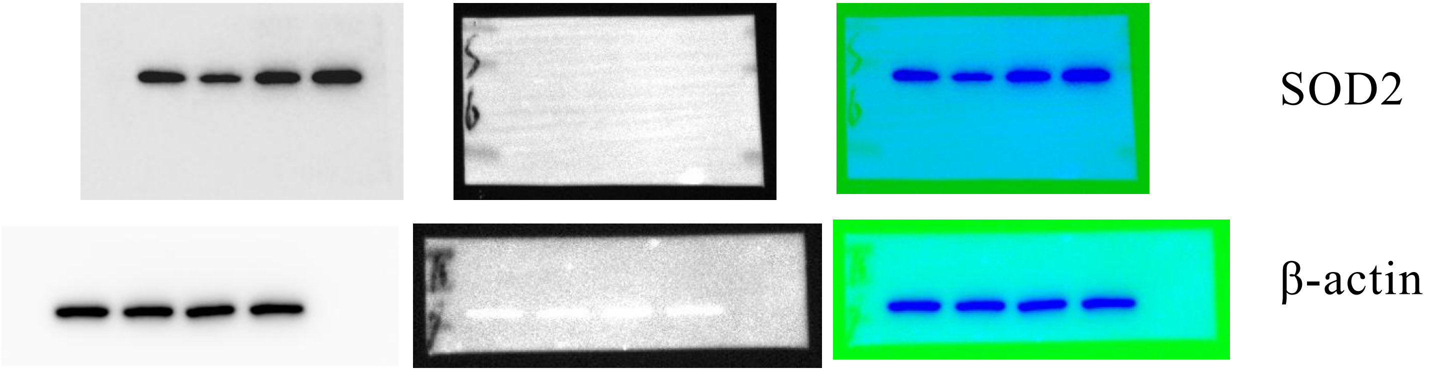

Fig.4A Immunoblotting analysis of the protein expression of SOD2

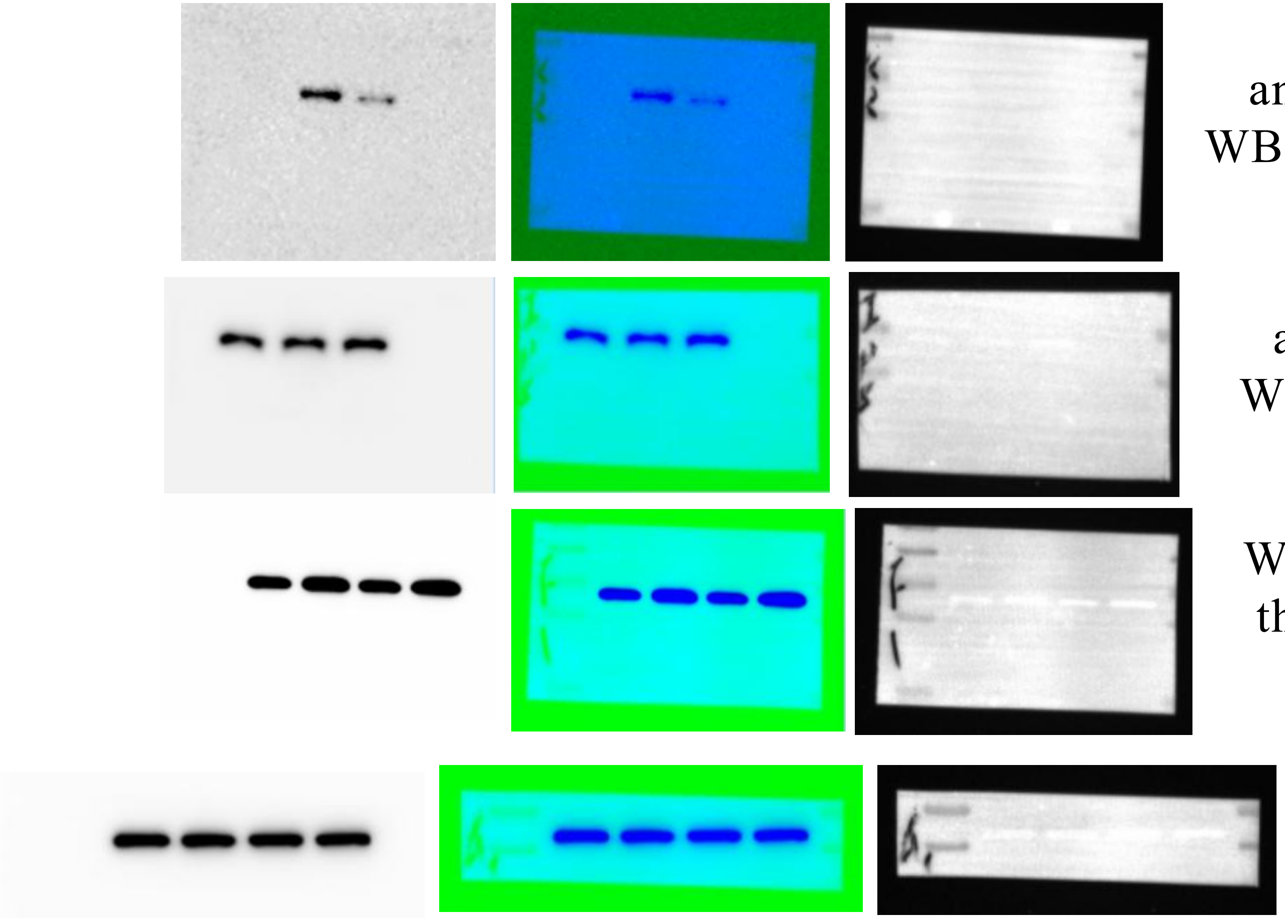

anti-Flag bead IP
WB analysis of Kbhb

anti-Flag bead IP
WB analysis of Flag

WB analysis of Flag in
the whole-cell lysates
(input)

WB analysis of β-actin
in the whole-cell
lysates (input)

Fig.4D J774A.1 macrophages were transfected with the indicated plasmids and treated with or without 5 mM β-OHB for 24 h, followed by IP using anti-Flag beads and immunoblotting analysis of SOD2 Kbhb levels

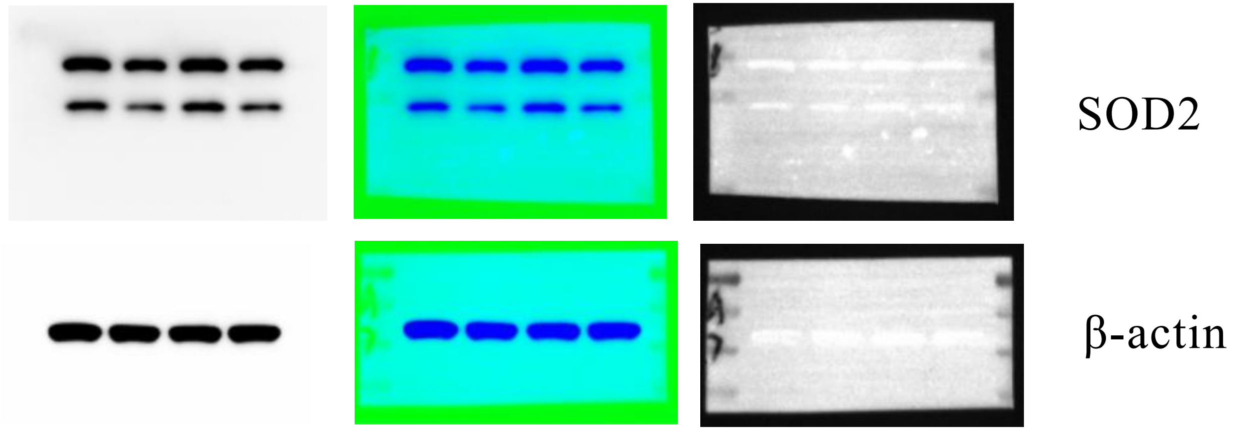

Fig.4F Immunoblotting analysis of the protein expression of SOD2

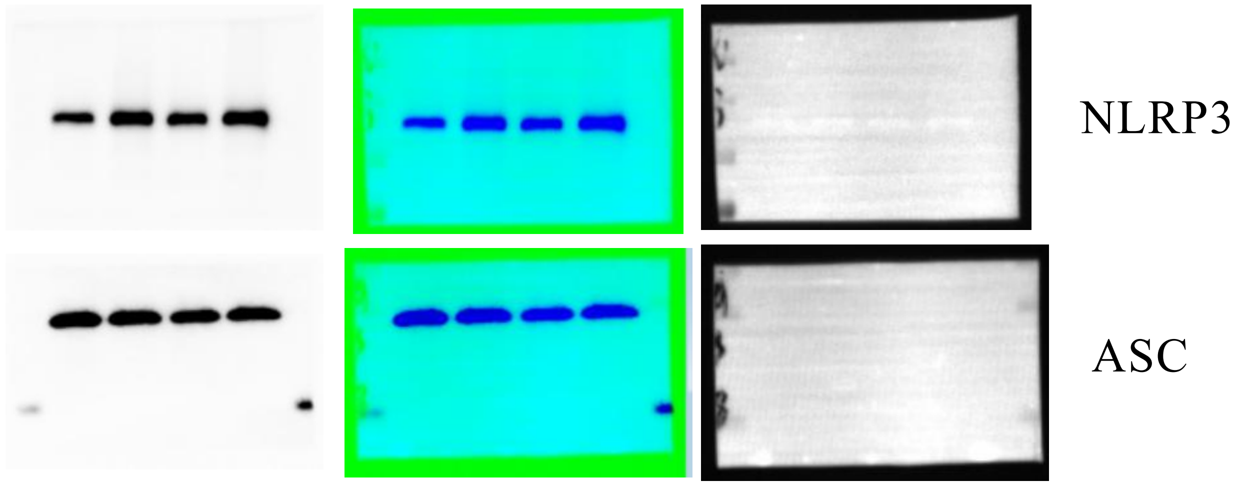

Fig.4L Immunoblotting analysis of the protein expression of NLRP3, ASC and cleaved-caspase 1

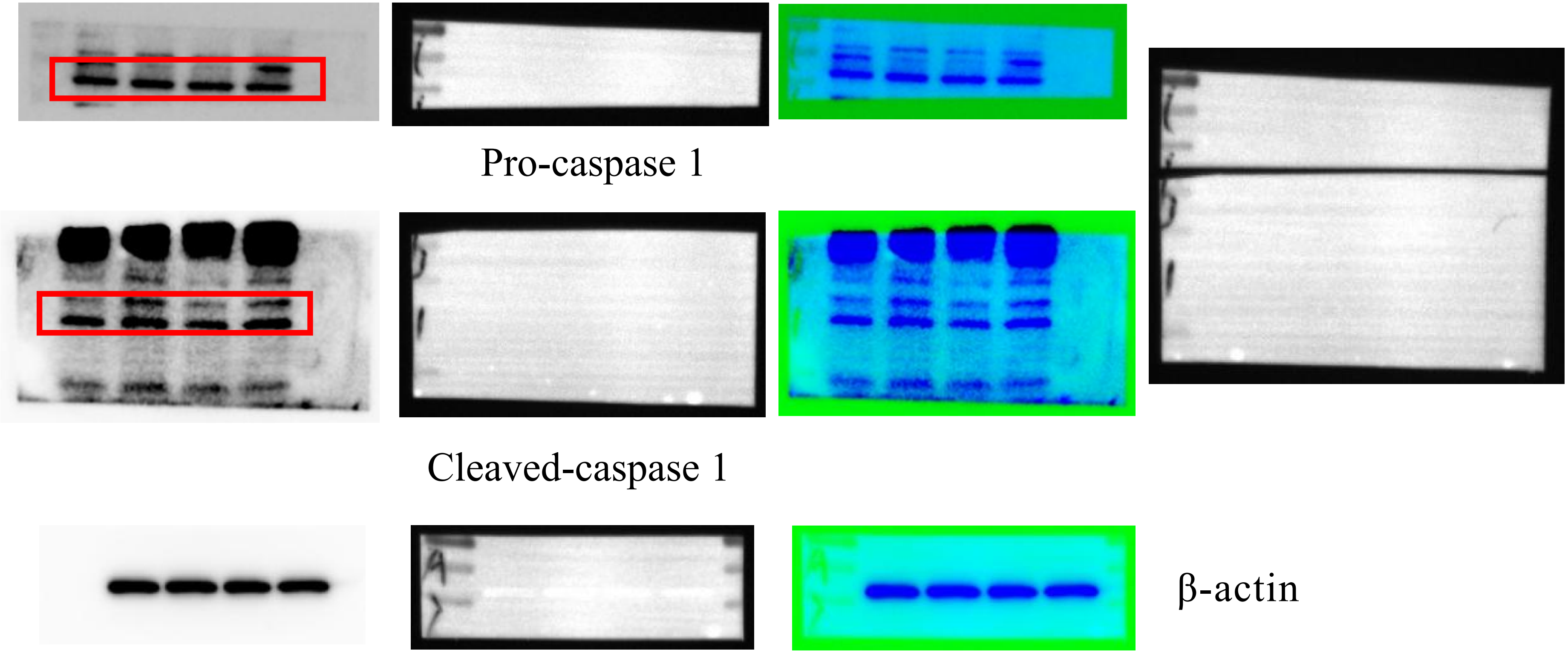

Fig.4L Immunoblotting analysis of the protein expression of NLRP3, ASC and cleaved-caspase 1

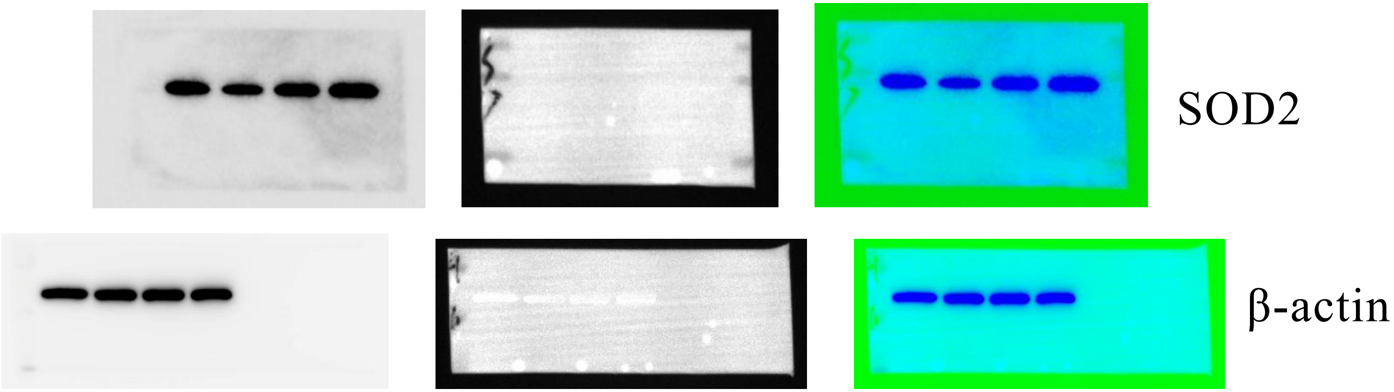

Fig.5A Immunoblotting analysis of the protein expression of SOD2

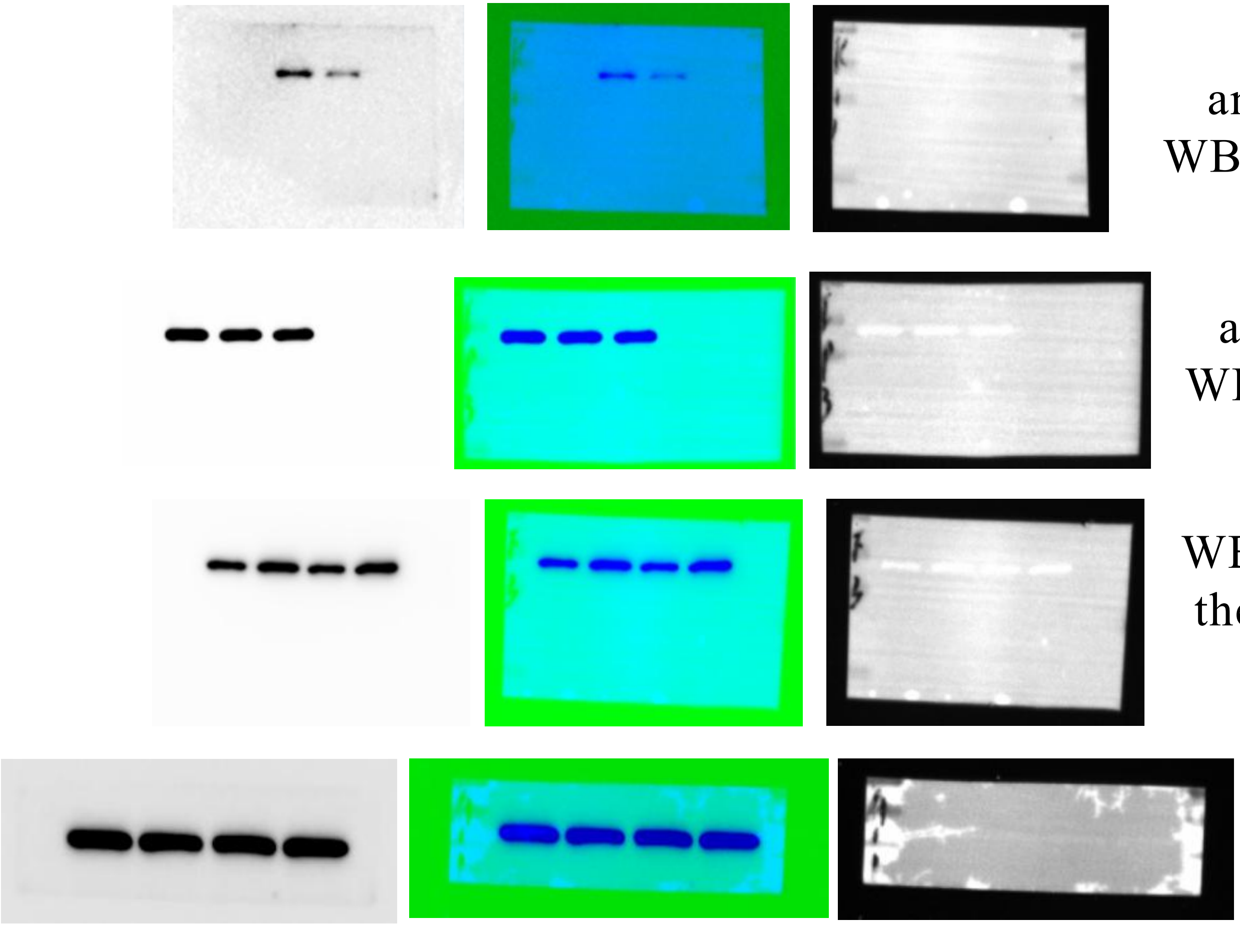

anti-Flag bead IP
WB analysis of Kbhb

anti-Flag bead IP
WB analysis of Flag

WB analysis of Flag in the whole-cell lysates (input)

WB analysis of β-actin in the whole-cell lysates (input)

Fig.5D AML12 hepatocytes were transfected with the indicated plasmids and treated with or without 5 mM β-OHB for 24 h, followed by IP using anti-Flag beads and immunoblotting analysis of SOD2 Kbhb levels

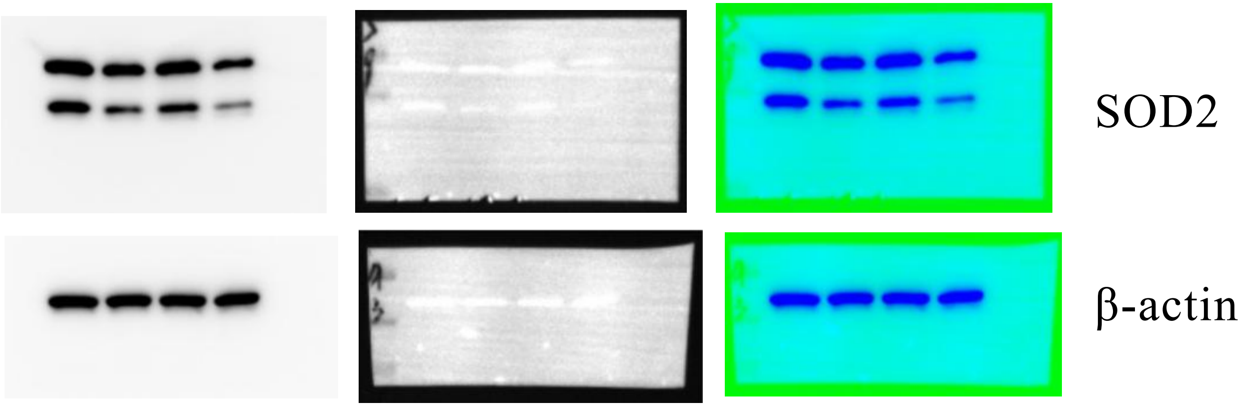

Fig.5F Immunoblotting analysis of the protein expression of SOD2

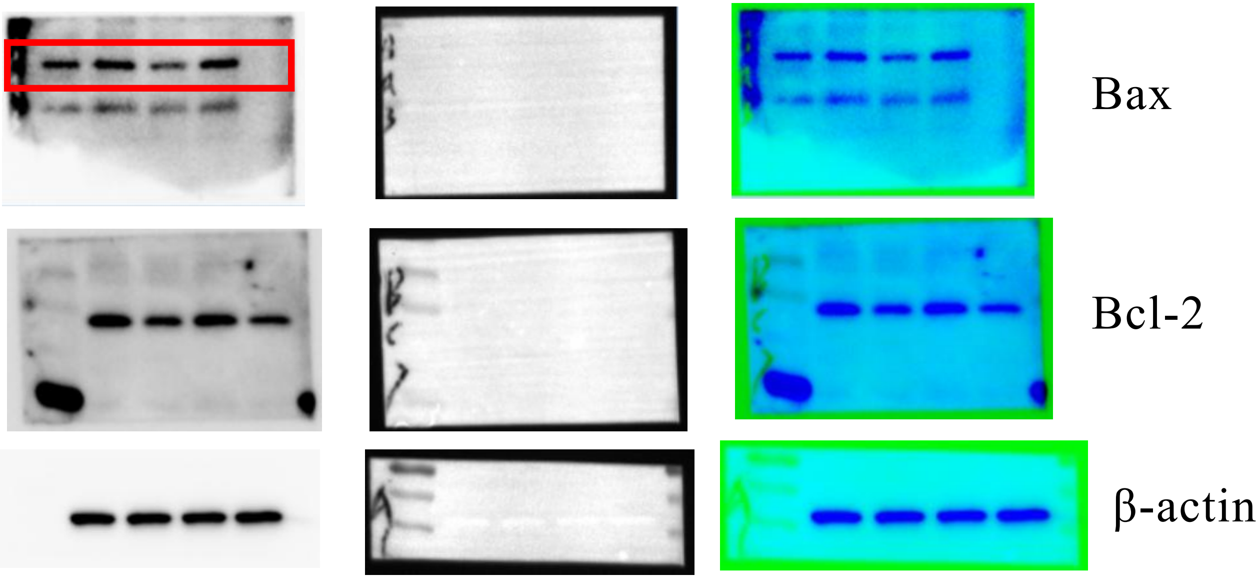

Fig.5O Immunoblotting analysis of the protein expression of Bax and Bcl-2

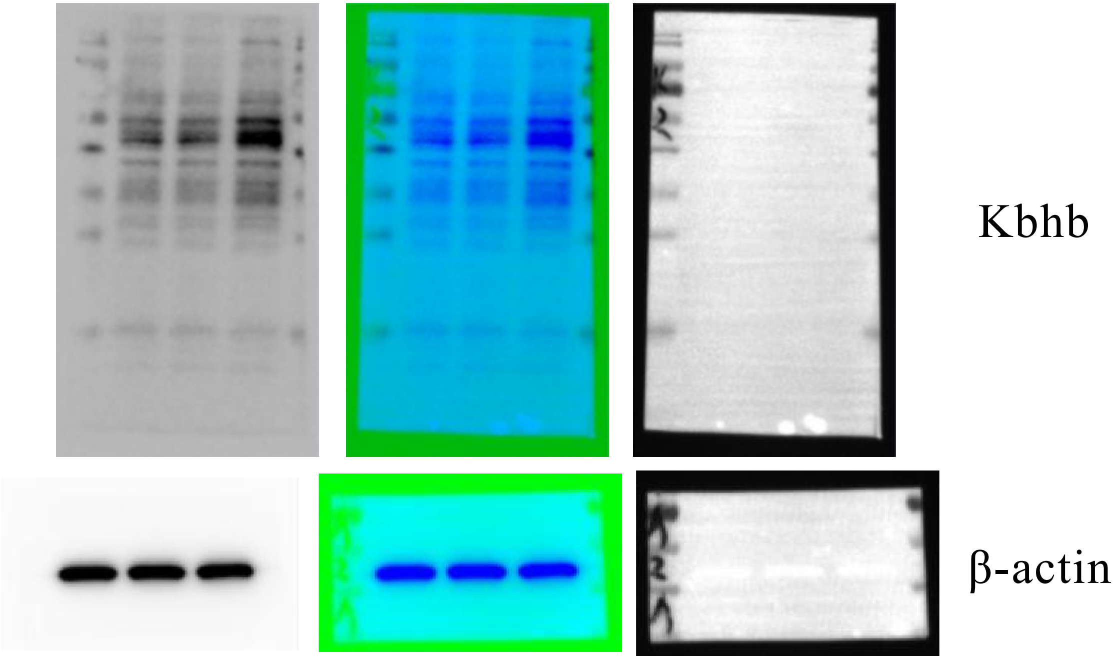

Fig.6E Immunoblotting analysis of the Kbhb modification in the hepatic tissues

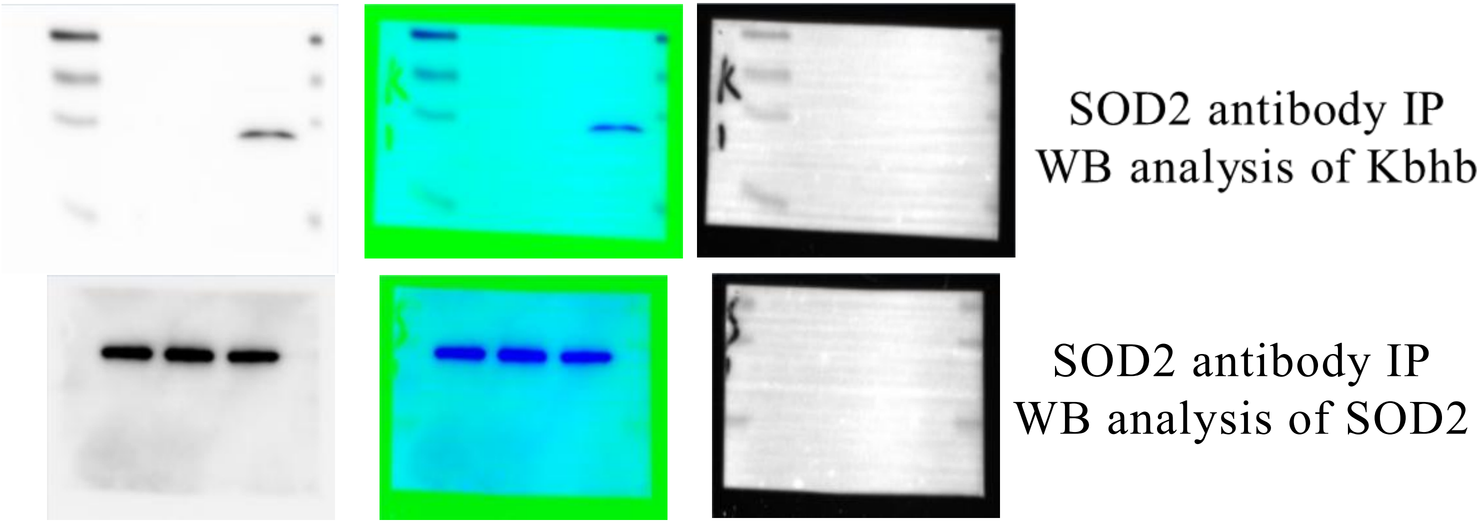

Fig.6F After IP using anti-SOD2 antibodies, immunoblotting analysis of the SOD2 protein expression and its Kbhb levels in hepatic tissues was performed

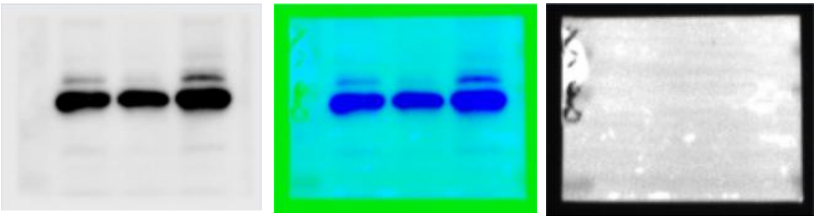

WB analysis of SOD2 in the whole-cell lysates (input)

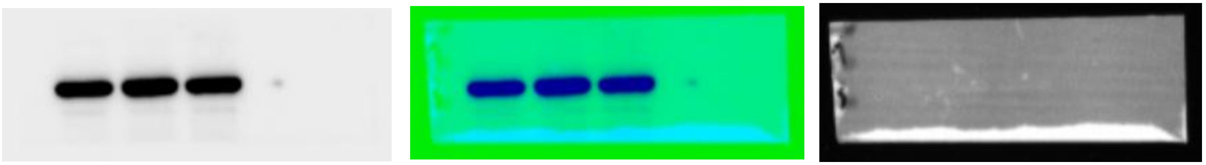

β-actin

Fig.6F After IP using anti-SOD2 antibodies, immunoblotting analysis of the SOD2 protein expression and its Kbhb levels in hepatic tissues was performed

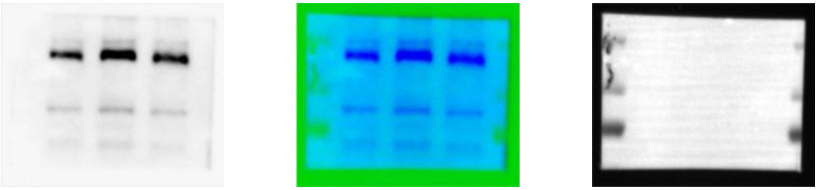

NLRP3

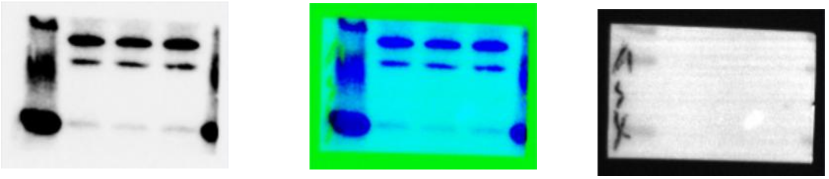

ASC

Fig.6I Immunoblotting analysis of the hepatic NLRP3, ASC and cleaved-caspase 1 protein expression

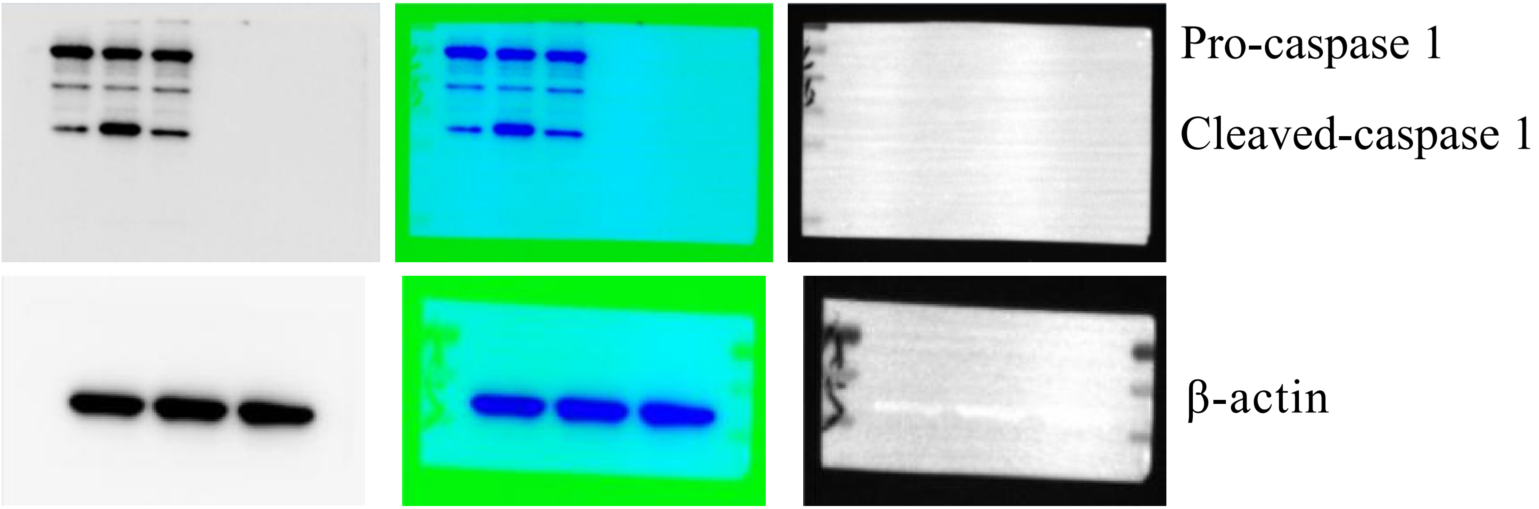

Fig.6I Immunoblotting analysis of the hepatic NLRP3, ASC and cleaved-caspase 1 protein expression

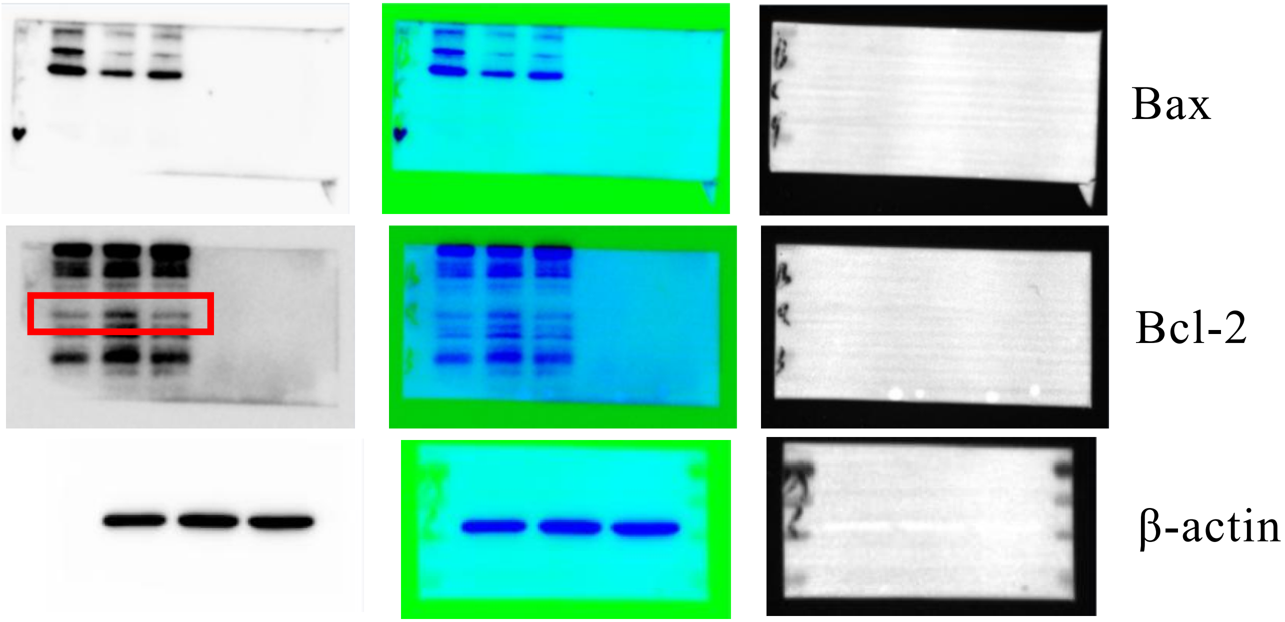

Fig.6N Immunoblotting analysis of Bax and Bcl-2 protein expression in the hepatic tissues

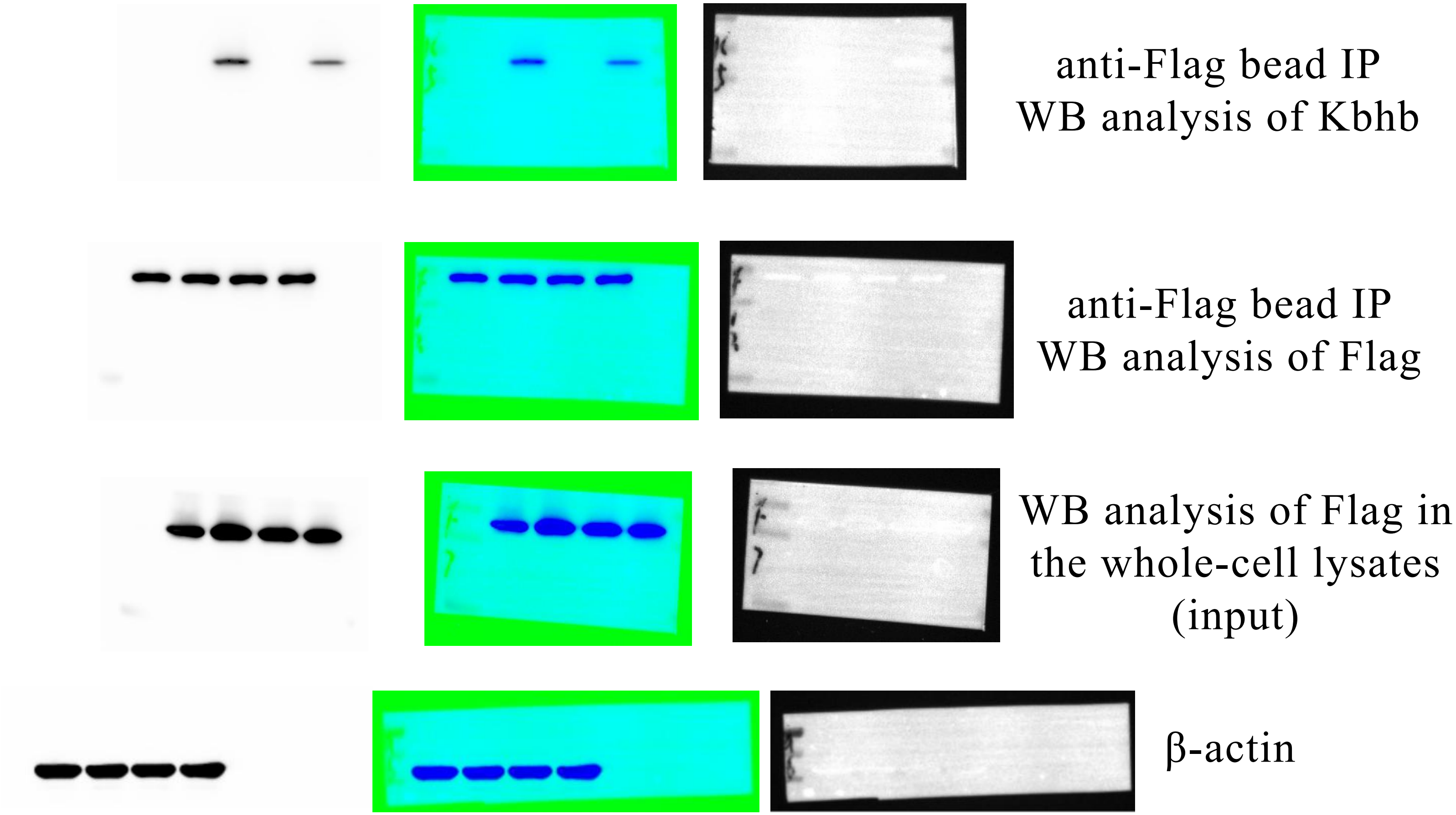

Fig.7E After IP using anti-Flag beads, immunoblotting analysis of the Flag-tagged protein and its Kbhb levels in hepatic tissues was performed

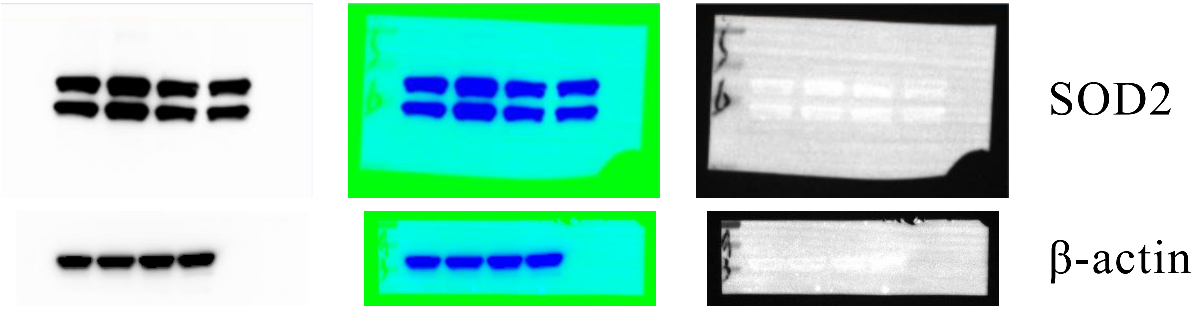

Fig.7G Immunoblotting analysis of the SOD2 protein expression in the hepatic tissues

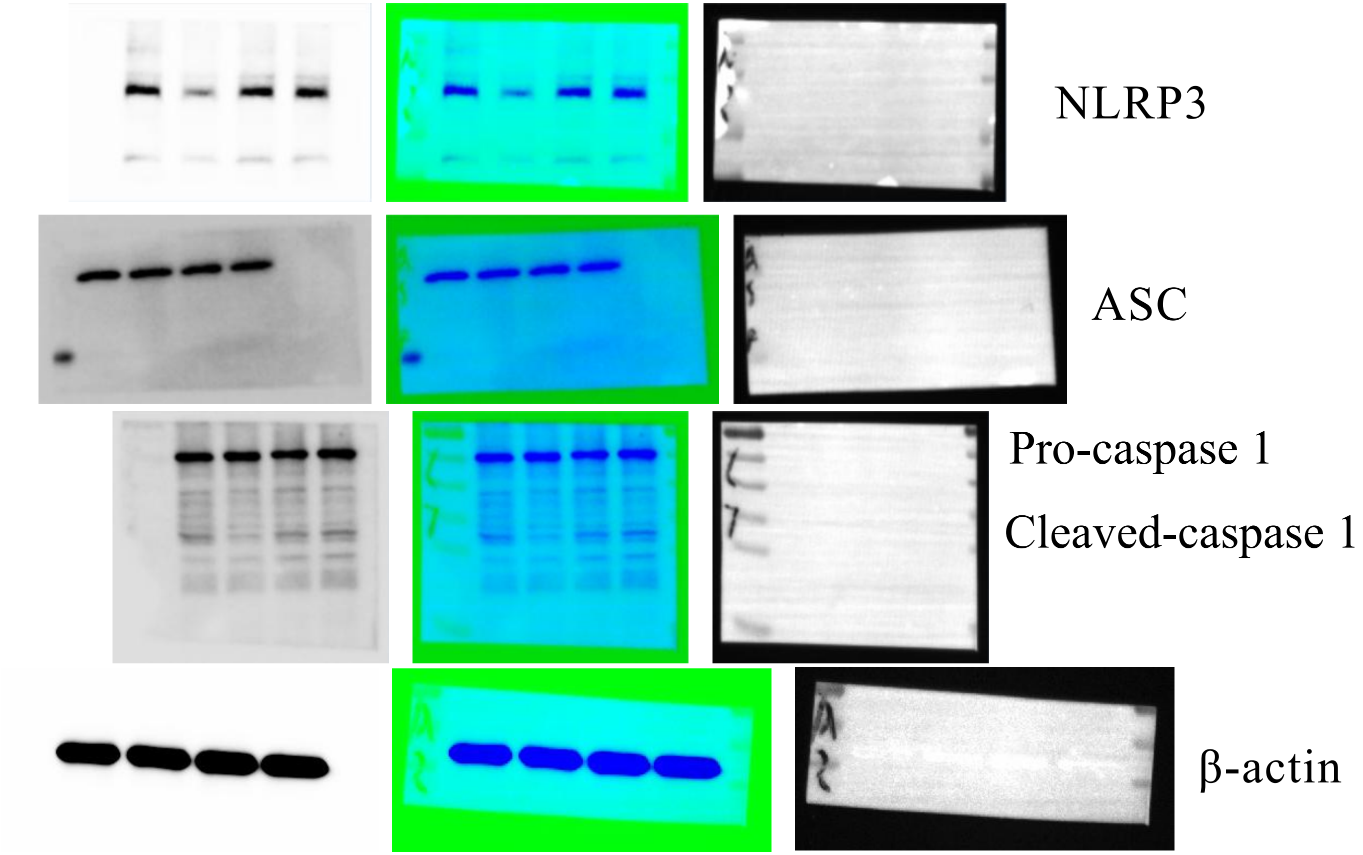

Fig.7J Immunoblotting analysis of the hepatic NLRP3, ASC and cleaved-caspase 1 protein expression

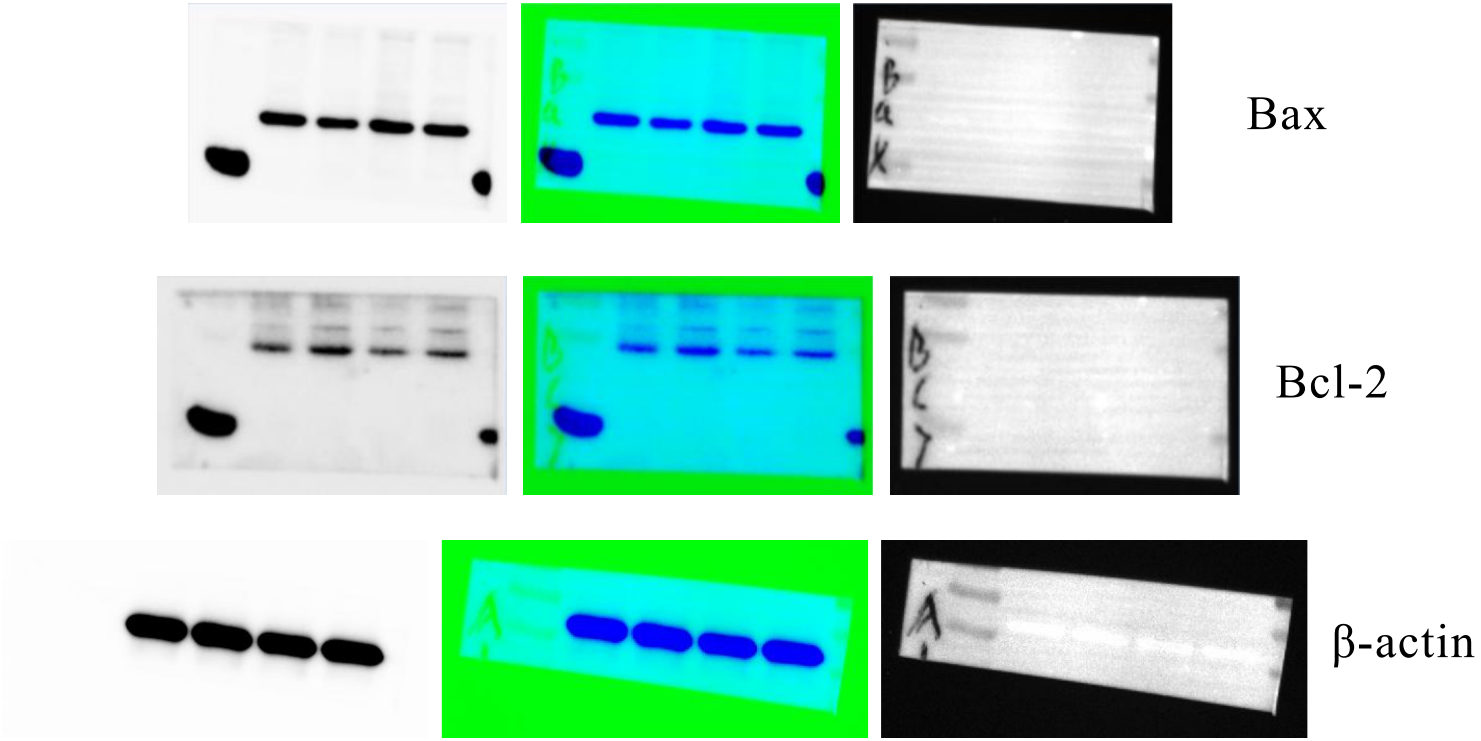

Fig.7O Immunoblotting analysis of Bax and Bcl-2 protein expression in the hepatic tissues

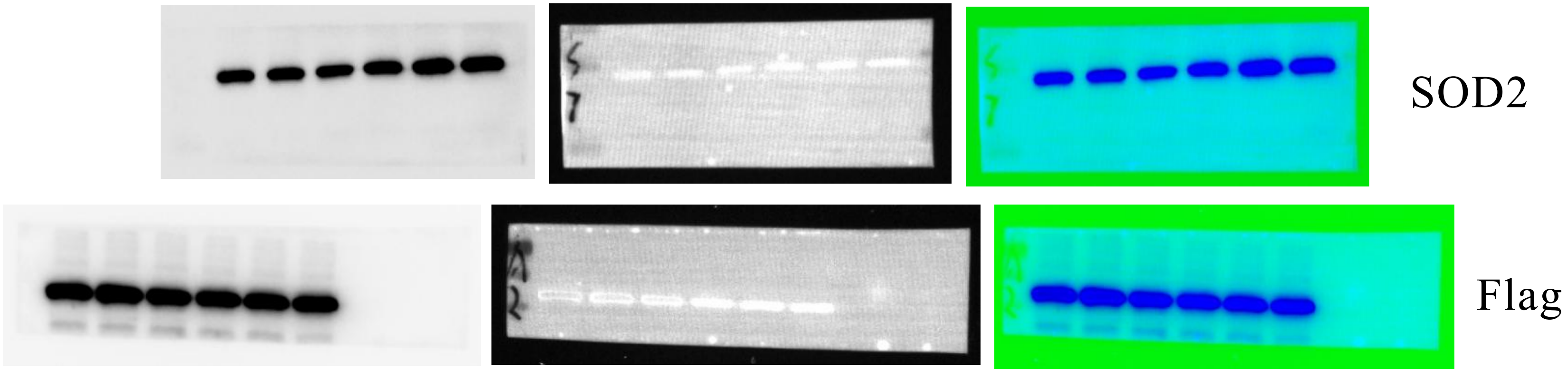

SFig.2A HEK293T cells were treated with or without 10 μM MG132 for 12 h. Immunoblotting analysis of the protein expression of SOD2

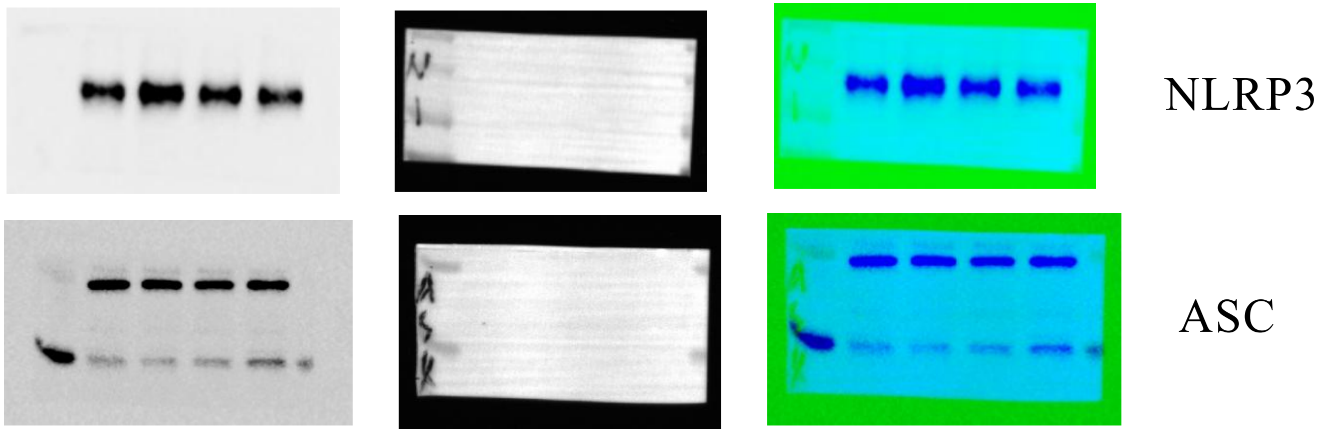

SFig.3D Immunoblotting analysis of the protein expression of NLRP3, ASC and cleaved-caspase 1

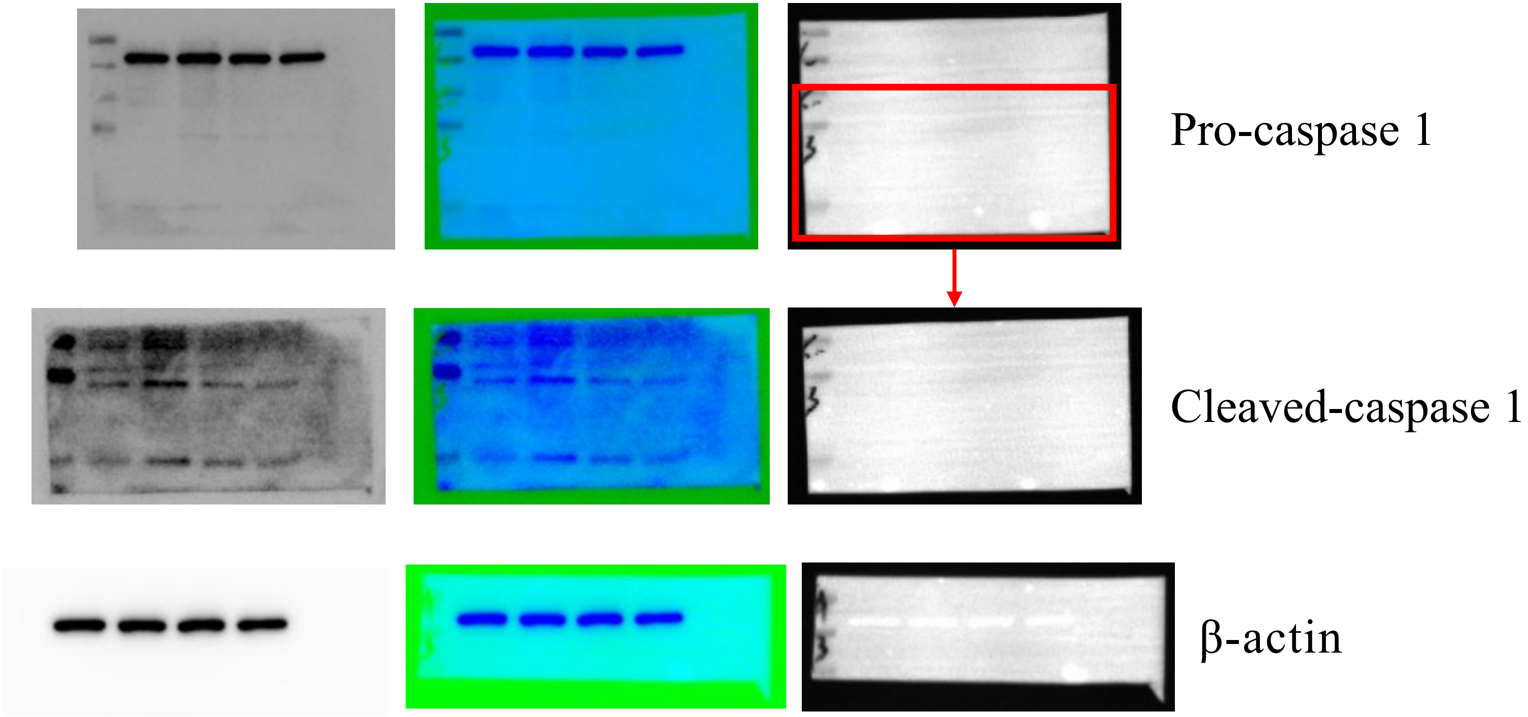

SFig.3D Immunoblotting analysis of the protein expression of NLRP3, ASC and cleaved-caspase 1

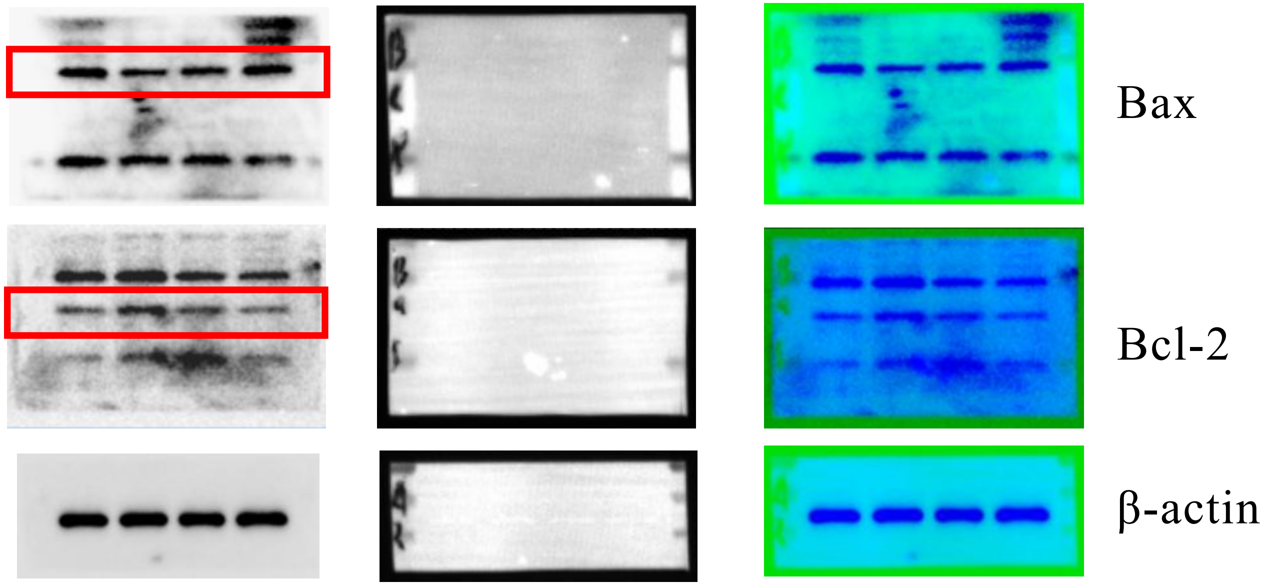

SFig.4G Immunoblotting analysis of the protein expression of Bax and Bcl-2
